# User-Centered Design of a Gamified Mental Health App for Adolescents in Sub-Saharan Africa: Multicycle Usability Testing Study

**DOI:** 10.2196/51423

**Published:** 2023-11-30

**Authors:** Julia R Pozuelo, Bianca D Moffett, Meghan Davis, Alan Stein, Halley Cohen, Michelle G Craske, Meriam Maritze, Princess Makhubela, Christine Nabulumba, Doreen Sikoti, Kathleen Kahn, Tholene Sodi, DoBAt & Ebikolwa Consortium, Alastair van Heerden, Heather A O’Mahen

**Affiliations:** 1 Department of Global Health and Social Medicine Harvard Medical School Harvard University Boston, MA United States; 2 Department of Psychiatry University of Oxford Oxford United Kingdom; 3 MRC/Wits Rural Public Health and Health Transitions Research Unit (Agincourt) School of Public Health, Faculty of Health Sciences University of the Witwatersrand Johannesburg South Africa; 4 Mind Ease London United Kingdom; 5 Africa Health Research Institute KwaZulu Natal South Africa; 6 Lincoln College University of Oxford Oxford United Kingdom; 7 Department of Psychology University of California, Los Angeles Los Angeles, CA United States; 8 Department of Psychiatry and Biobehavioral Sciences University of California, Los Angeles Los Angeles, CA United States; 9 BRAC Kampala Uganda; 10 Umeå Centre for Global Health Research, Division of Epidemiology and Global Health Department of Public Health and Clinical Medicine Umeå University Umeå Sweden; 11 SAMRC-DSI/NRF-UL SARChI Research Chair in Mental Health and Society University of Limpopo Limpopo South Africa; 12 See Acknowledgements; 13 Center for Community Based Research Human Sciences Research Council Pietermaritzburg South Africa; 14 SAMRC/Wits Developmental Pathways for Health Research Unit, Department of Paediatrics School of Clinical Medicine, Faculty of Health Sciences University of the Witwatersrand Johannesburg South Africa; 15 Mood Disorders Centre Department of Psychology University of Exeter Exeter United Kingdom

**Keywords:** depression, adolescents, mental health app, behavioral activation, user-centered design, low- and middle-income countries, mobile phone

## Abstract

**Background:**

There is an urgent need for scalable psychological treatments to address adolescent depression in low-resource settings. Digital mental health interventions have many potential advantages, but few have been specifically designed for or rigorously evaluated with adolescents in sub-Saharan Africa.

**Objective:**

This study had 2 main objectives. The first was to describe the user-centered development of a smartphone app that delivers behavioral activation (BA) to treat depression among adolescents in rural South Africa and Uganda. The second was to summarize the findings from multicycle usability testing.

**Methods:**

An iterative user-centered agile design approach was used to co-design the app to ensure that it was engaging, culturally relevant, and usable for the target populations. An array of qualitative methods, including focus group discussions, in-depth individual interviews, participatory workshops, usability testing, and extensive expert consultation, was used to iteratively refine the app throughout each phase of development.

**Results:**

A total of 160 adolescents from rural South Africa and Uganda were involved in the development process. The app was built to be consistent with the principles of BA and supported by brief weekly phone calls from peer mentors who would help users overcome barriers to engagement. Drawing on the findings of the formative work, we applied a narrative game format to develop the Kuamsha app. This approach taught the principles of BA using storytelling techniques and game design elements. The stories were developed collaboratively with adolescents from the study sites and included decision points that allowed users to shape the narrative, character personalization, in-app points, and notifications. Each story consists of 6 modules (“episodes”) played in sequential order, and each covers different BA skills. Between modules, users were encouraged to work on weekly activities and report on their progress and mood as they completed these activities. The results of the multicycle usability testing showed that the Kuamsha app was acceptable in terms of usability and engagement.

**Conclusions:**

The Kuamsha app uniquely delivered BA for adolescent depression via an interactive narrative game format tailored to the South African and Ugandan contexts. Further studies are currently underway to examine the intervention’s feasibility, acceptability, and efficacy in reducing depressive symptoms.

## Introduction

### Background

The incidence of depression peaks during adolescence (ages of 10-24 years), with the cumulative probability of depression rising from 5% in early adolescence to as high as 20% by the end of that period [1,2]. Left untreated, depression interferes with schooling and affects young people’s social relationships [2]. Depression in adolescence has been associated with a significant reduction in future income, greater risk of substance use, and risky sexual behaviors and is a major risk factor for suicide [3-5].

Depression is underdiagnosed and undertreated worldwide but particularly in low- and middle-income countries (LMICs) [6], where low levels of average public expenditure on mental health (<US $2 per capita) result in a significant shortage of mental health professionals and large treatment gaps [7]. For example, <5% of individuals needing treatment in LMICs receive minimally adequate treatment for depression [8]. In addition, high levels of mental health stigma prevent adolescents from seeking care [9]. These barriers, coupled with the devastating consequences of poverty, cause adolescents in LMICs to be disproportionately affected by the burden of depression.

With the increase in smartphone ownership and access to the internet, digital interventions such as smartphone apps are a promising strategy to reduce these large treatment gaps in LMICs [10,11]. This is especially relevant for adolescents given that they make up the vast majority of smartphone users [12]. Digital mental health interventions offer several advantages. They have the potential to be *delivered at scale* with low marginal costs for each additional user [13,14]. They may help *overcome stigma* as individuals can access them discreetly using their devices [15], and they are *flexible and convenient* as users can choose when, how, and where to access them. In LMICs, where individuals might have to travel long distances to access health care, the portability of digital technologies can save traveling time and reduce expenses. They may also help reduce the pressure on already overstretched health care systems [16]. Digital interventions open the possibility for more *tailored psychological treatments* without having to wait until the next appointment [17]. It also allows for continuous monitoring and prediction of suicide risk in real time [18]. Mental health apps may also *empower individuals* by making them feel more in control of their health and may help raise *societal awareness* of mental health issues [19,20].

Despite the many potential advantages of digital mental health interventions, evaluations of their effectiveness in adolescents have yielded mixed results [21,22]. Many studies are characterized by low adherence and high attrition rates [23,24]. For example, it is estimated that only 3.3% of users continue to engage with mental health apps after 30 days [24]. Although some commercial smartphone apps attract more users, many have not been rigorously evaluated and show little fidelity to evidence-based treatments [25,26]. Furthermore, most of the evidence has been gathered in high-income countries and, therefore, generalizability to an African context, where conditions and resources differ vastly, is questionable [10,21,27].

Given these limitations, this study used multiple user-centered and participatory action research methods to design and develop an app to address depression among adolescents in rural South Africa and Uganda. This paper documents the development process of the Kuamsha app (meaning *activate* in Swahili) and summarizes the results of multicycle usability testing.

### Treatment Model and Human-Supported App

We developed the app to be consistent with the principles of behavioral activation (BA)—a highly transferable and evidence-based treatment for adolescent depression—and to include human support (via phone calls), which has been associated with greater adherence and effectiveness of digital therapies [21,28]. We did not have any other preconceived ideas about the design or format of the app.

BA is an evidence-based psychological therapy derived from principles of response-contingent positive reinforcement given the evidence on low levels of such reinforcement in relation to depression. BA focuses on two core principles: (1) increasing activities that are meaningful and positively reinforcing for the individual (activation) and (2) addressing processes that inhibit activation (eg, negatively reinforced avoidance behaviors) [29].

BA was chosen as it is relatively simple to deliver, easily understood by patients, and less costly than cognitive behavioral therapy (one of the most researched forms of psychotherapy) [30,31]. BA has also been found to have similar effect sizes as antidepressant medications [32]. Furthermore, it has been effectively adapted for use with adolescents, in low-income and diverse cultural settings, and in digital formats [27,33-36].

Although BA was originally conceptualized as a treatment for depression, the underlying principles apply to broader populations as they include basic problem-solving skills for everyday challenges, awareness of how one’s behavior influences mood, reduction of avoidance behaviors, and redirecting attention away from ruminative thinking [29]. Indeed, BA has been recently proposed as a transdiagnostic approach, and there is evidence demonstrating its effectiveness in improving anxiety symptoms, activation [37], and overall well-being among the general population [37-39].

Although there are already some smartphone apps available that deliver BA therapy for depression (eg, Mobilyze!, MoodMission, Boost Me, Moodivate, and Behavioral Apptivation), none of these apps have been developed for adolescents in a sub-Saharan African context [40-44]. As such, these apps contain elements that are not feasible for use in low-resource settings: they are data intensive, are only available in Apple’s iOS platforms for use on iPhones (which are expensive), or are intended to be used in conjunction with a licensed clinician.

To improve adherence, the Kuamsha app was built to be supported by brief weekly phone calls from peer mentors. Although only a handful of studies have explored the involvement of lay workers in digital mental health interventions [35], task shifting using nonspecialist workers has been increasingly used in sub-Saharan Africa to deliver physical and mental health treatment services [45,46]. We designed the app to be supported by trained lay workers who would serve as peer mentors and whose main role was limited to helping users understand the app’s content and overcome barriers to engagement. The peer mentor component was designed through an iterative process involving a multidisciplinary group of mental health professionals, public health specialists, and researchers from Uganda, South Africa, the United Kingdom, and the United States. We drew from established models of peer-to-peer coaching [34,47,48] and iteratively adapted the program to ensure that it was culturally relevant. The details of the training and supervision of these peer mentors will be described in a separate study.

## Methods

### Ethical Considerations

The study was reviewed and approved by institutional review boards in South Africa (the University of the Witwatersrand [Wits] Human Research Ethics Committee [M181027], Ehlanzeni District, and Mpumalanga Provincial Departments of Health and Education [MP_201903_003]), Uganda (Makerere University School of Public Health [HDREC 750] and the Uganda National Council for Science and Technology [UNCST HS724ES]), and the United Kingdom (Oxford Tropical Research Ethics Committee, OxTREC 39-18).

Written informed consent was obtained from either the parent or legal guardian of adolescents aged 15 to 17 years or directly from adolescents aged ≥18 years. In addition, written assent was obtained from participants aged <18 years.

To protect the participants’ confidentiality, adolescents were identified only by a participant ID number. The interviews were audio recorded and transcribed verbatim, removing any potentially identifiable information. Electronic audio recordings were destroyed once transcribed. Identifying information (name, address, and contact information) was stored separately from all other data in a secure and locked space within the South Africa and Uganda offices. Access to this information was strictly limited to specific named project staff members.

Participants were not paid for taking part in the study, although they were provided with light refreshments (juices and snacks) following the interviews. Participants in the prepilot were provided with a smartphone, which they could keep at the end of the study. No other incentives or benefits were offered.

The app was developed as part of the “DoBat and Ebikolwa n’empisa” research programs, which aim to develop scalable psychological treatments to address depression among adolescents in low-resource settings. The trial in South Africa was registered with the South African National Clinical Trials Register (DOH-27-112020-5741) and the Pan African Clinical Trials Registry (PACTR202206574814636).

### Study Setting

**Figure 1:**
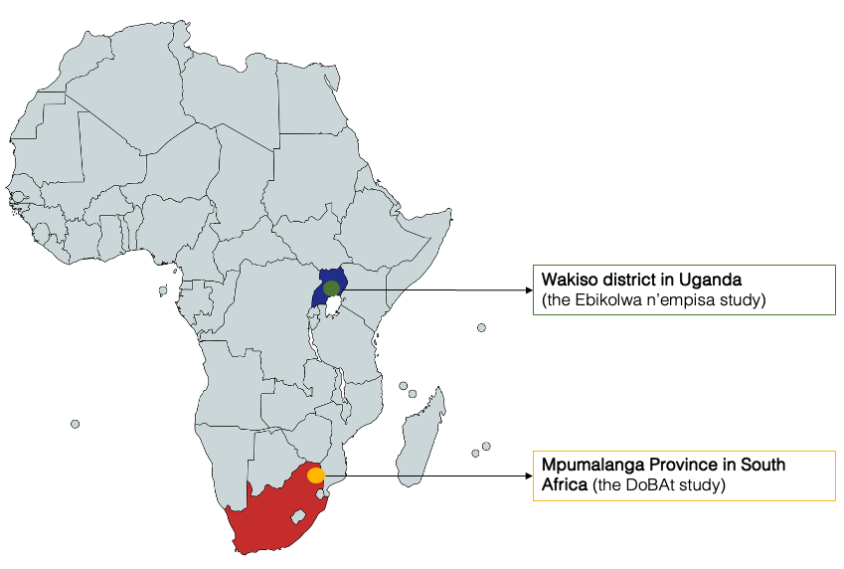
Study locations in South Africa and Uganda.

The study was conducted in 2 different countries and settings, each representing a different cultural and social context (Figure 1). In South Africa, the study was embedded in the Medical Research Council and Wits Rural Public Health and Health Transitions Research Unit Agincourt health and sociodemographic surveillance system study area in the rural Bushbuckridge subdistrict of Mpumalanga Province, South Africa. The study area is located along the western border of Mozambique and comprises 31 adjacent villages with a population of 117,000 in 21,000 households [49]. As in other parts of the country, there is a high prevalence of depression among adolescents (18.2%) and a paucity of mental health and social care services, particularly psychological therapists [50-52].

In Uganda, the study was carried out in the Wakiso District, a periurban area located approximately 30 km from Kampala. The Wakiso District is home to nearly 2 million people. The site combines periurban communities close to the Entebbe-Kampala road with relatively isolated fishing villages extending to the shores of Lake Victoria. The study area is poor, and the most common occupation is subsistence farming. Among adolescents, 50% attend secondary school, and 15% of youth (aged 18-30 years) are neither working nor in school. The nearest specialist psychiatric resources are in Kampala, a 2-hour journey by public transportation [53].

In both settings, mobile phone ownership is on the rise. A 2019 census conducted at the study site in South Africa showed that >93% of households owned a cell phone and 82% had a smartphone. Data from the 2014 National Population and Housing Census in Uganda suggest that the proportion of adolescents who own mobile phones in the Wakiso District is >80% and increasing steadily [53].

### Study Participants

The study’s target population was adolescents aged between 15 and 19 years from South Africa and Uganda. We focused on midadolescence specifically as it is a transitional period when individuals start to develop their self-identity and make vital decisions within the context of less parental control and heightened peer influence [1]. It is also a time when the prevalence of depression increases significantly, accounting for more disability-adjusted life years than any other mental health condition [54]. Furthermore, adolescents within this age range are more likely to be familiar with smartphones than their younger counterparts, enhancing the practical feasibility of our intervention.

To be eligible to participate, respondents had to be residents of the study sites; be fluent in Xitsonga (South Africa), Luganda (Uganda), or English (either country); be willing and able to provide informed consent or assent; and have a caregiver willing to provide consent (if aged <18 years). We aimed to achieve a balance of sex and age representation in our sample.

Other recruited stakeholders included caregivers of adolescents, schoolteachers, community stakeholders, and mental health care providers. The objective of these discussions was to elicit various views and perspectives from multiple stakeholders, triangulate these perspectives with the information gathered from adolescents, and ensure that the intervention was culturally acceptable within communities.

Different recruitment strategies were followed across study settings. In South Africa, the local partner (the Medical Research Council and Wits-Agincourt Research Unit) consulted the Community Advisory Board and key educational stakeholders to obtain their advice and approval for the project. Adolescents in South Africa were recruited via their schools. In Uganda, the local partner (BRAC Uganda) first obtained verbal approval from the local council chairperson and the community elders before beginning recruitment. Adolescents in Uganda were recruited via the BRAC network of clubs for adolescents (Empowerment and Livelihood for Adolescents [ELA] clubs). Field supervisors visited the schools (South Africa) and ELA clubs (Uganda) and provided an overview of the study and written information sheets for participants and parents or guardians. Adolescents willing to participate in the study were asked to return their signed assent or consent forms to a field-worker who collected them at school or ELA clubs on another occasion. Owing to the challenges of the COVID-19 pandemic and limited resources, convenience sampling was deemed to be the most appropriate sampling method.

### Study Design and Development Phases

#### Overview

An iterative user-centered agile design approach was used to co-design the app with the study’s target population [55-57]. An array of qualitative research methods was used, including focus group discussions, in-depth interviews, participatory workshops, usability testing sessions, questionnaires, and expert consultation. Discussions were framed using various elicitation techniques, including semistructured topic guides, role-playing exercises, and card-sorting games.

User-centered development was carried out in 4 phases: conceptualization, prototyping, product release, and evaluation. An iterative feedback loop approach was used in which each phase’s findings were used as inputs for the following phases and to refine previous phases. Table 1 summarizes the development stages.

The objectives and types of elicitation techniques used in each phase are outlined in the following sections. Samples of interview guides and elicitation techniques are provided in Multimedia Appendix 1.

**Table 1:** Development stages.

Stage and method		Sample size, n	Country	Date
**Conceptualization**				
	5 focus groups with adolescents	37	South Africa	July 2019-August 2019
	In-depth interviews with 4 adolescents from the focus groups	4	South Africa	October 2019
	2 focus groups with caregivers (n=6) and schoolteachers (n=7)	13	South Africa	December 2019-January 2020
**Prototyping**				
	8 usability testing sessions to assess app features	8	South Africa	February 2020-March 2020
	3 focus groups to test Kuamsha wireframes	34	Uganda	August 2020
	6 participatory workshops	40	South Africa and Uganda	September 2020-January 2021
**Product release**				
	24 usability testing sessions with the Kuamsha app	24	South Africa and Uganda	February 2021
	Prepilot study with adolescents with depression	17	South Africa	December 2021-May 2022
**Evaluation**				
	Feasibility studies in Uganda (n=31) and South Africa (n=196)	227	South Africa and Uganda	Results expected in late 2023

#### Conceptualization Phase

The objective of this phase was to understand adolescents’ needs and context, assess the feasibility of delivering BA using a digital platform, and decide on the app’s main features. This phase included 5 focus groups with adolescents, 4 in-depth interviews with adolescents who provided helpful feedback in the focus groups, and 2 focus groups with caregivers and schoolteachers. The purpose of these discussions was to (1) discuss adolescents’ values, goals, struggles, social relationships, and the type of meaningful activities they engage in; (2) learn about access to and use of smartphones and desirable content and features in apps; (3) examine perceptions and local language terms for depression and understand the local support systems; and (4) understand caregivers’ and teachers’ views on the potential limitations of using digital tools in this population.

#### Prototyping Phase

The objective of this phase was to use the results from phase 1 to develop a simple, scaled-down version of the app and ask adolescents to provide feedback. This phase included 8 usability testing sessions, 3 focus groups, and 6 participatory workshops. In the usability testing sessions, adolescents were given smartphones and asked to interact with apps that targeted different app components (eg, mood monitoring). Facilitators observed their behavior and asked questions as they progressed. During the focus groups, paper-based wireframes and basic prototypes of the Kuamsha app were presented, and adolescents were asked to provide feedback on the app features, including story characters, artwork, background music, and game elements. The participatory workshops were all-day events in which adolescents and other stakeholders interacted with and provided additional feedback on the app’s content.

#### Product Release Phase

On the basis of the outcomes of phases 1 and 2, the objective of this phase was to build a minimum viable product (MVP) and test it with the study’s target population. This phase included 24 usability testing sessions in which adolescents interacted with the Kuamsha app and were asked to provide feedback on usability, design, and recommendations for improvements. Some of these sessions were unguided, in which adolescents were asked to freely explore the app and verbalize their experiences and general impressions (think-aloud methodology) [58]. This method was helpful in investigating whether the user interface was easy to use without much explanation. Facilitators were trained to take notes on how the app was explored, including features that adolescents did not interact with. Other sessions were guided, in which the facilitator walked participants through each screen while probing for understanding. This phase also included a prepilot study with 17 adolescents with depression in South Africa. The entire 11-week intervention was tested, including the peer mentor component.

#### Evaluation Phase

This phase included 2 studies that explored the intervention’s feasibility, acceptability, and efficacy in reducing depressive symptoms. One was a feasibility study in Uganda with 31 adolescents from the general population (the *Ebikolwa n’empisa study*). The other was a randomized controlled pilot trial in South Africa with 196 adolescents with depression (the *DoBAt study*) [59]. The results from both of these studies are expected in late 2023.

### Sampling Procedures Across Development Phases

The sample sizes for the conceptualization, product release, and evaluation phases were predetermined and aligned with our a priori research design. In contrast, we maintained a more flexible approach when it came to the prototyping phase. This variability was driven by the dynamic and iterative nature of the app development process. Specifically, the sample size of the usability testing sessions was adjusted with each app refinement or whenever concerns about specific app features arose. We attempted to reach saturation throughout each design stage before advancing to the next development phase.

### Analysis

Descriptive statistics summarized the participants’ characteristics. Interviews and focus group discussions were audio recorded and transcribed verbatim, removing any potentially identifiable information to protect participants’ privacy. Transcribed interviews were summarized and grouped into main themes using the framework method [60].

Data from the workshops and usability testing sessions were analyzed using an instant data analysis approach extensively used in the development of technologies [61]. Under this approach, the interviewers were trained to record all problems that arose during the sessions. Following these sessions, the interviewers and a group of study investigators discussed and ranked these problems. This technique reduces the time required for transcribing and analyzing interviews and allows for the fast identification of the most critical and severe usability problems.

The evidence from all development phases was triangulated to develop a game design document that outlined the platform’s main features and “guiding principles.” This document was used as a road map for the development of the app.

## Results

In this section, we summarize the main findings of each developmental phase and describe how the user-centered design approach informed specific app components.

### Conceptualization Phase

#### Overview

In total, 50 individuals were involved in this phase: 37 (74%) adolescents, 6 (12%) caregivers, and 7 (14%) schoolteachers from the study site in South Africa.

Adolescents who took part in the focus group discussions were aged 17.1 (SD 1.42) years on average, and the sample was evenly split between male and female participants. All adolescents were enrolled in school and had an average 9.7 (SD 1.27) years of schooling. On average, about one-third (11/37, 30%) had part-time jobs, including selling products in the market, cutting firewood, herding cattle, gardening, and working as a hairdresser. Most adolescents (33/37, 89%) had access to a smartphone.

Study participants were very supportive of the idea of having an app that would support their mental health:

I like the idea of getting advice through an app because I would get the help I need, unlike if I were to get advice from a person.

P1; female; focus group 4

I like the idea because there are times when you don’t feel like talking to anyone, or you don’t have someone to talk to, so the app would be a good idea.

P5; male; usability testing session 4

Adolescents’ preferences regarding a mental health app revolved around 4 main themes: app features, cultural validity, confidentiality, and technological aspects. An excerpt of the results is presented in Table 2.

Caregivers and schoolteachers supported the idea of an app-based intervention to address depression among adolescents, which they acknowledged as an unaddressed problem. They also expressed their concerns about adolescents’ tendency to overuse and misuse their phones (eg, accessing inappropriate content). These findings helped frame how the intervention was conducted but did not directly feed into the app’s design. For example, the insights from caregivers and schoolteachers influenced our decision to perform in-school recruitment in South Africa rather than through household visits. Furthermore, they emphasized the importance of addressing adolescents’ phone use habits, resulting in the development of user guides and measures to limit data use. The results of these focus groups will be published with the results of the main trial.

**Table 2:** Conceptualization phase: excerpt of adolescents’ preferences regarding app components.

Theme and subtheme			Quotes
**App features**			
	Use of stories	“The app needs to have pictures of people who have been through difficult situations and how they managed to get out of those situations.” [P3; male; FG^a^ 2] “[The app] must include pictures or videos of people who had depression before.” [P7; female; FG 1] “[The app] could display a person feeling low and having their friend around.” [P2; female; FG 4] “I love the comic videos that I can download on my phone and watch stories.” [P2; male; FG 2]	
	Gamification	“Getting points will motivate us to go back to the game and play it every day.” [P1; female; FG 4] “If we find that we are getting help it should take us to another level.” [P8; female; FG 1] “Personalising your character helps you to understand yourself and know yourself better.” [P3; female; FG 5]	
**Cultural validity**			
	Esthetics	“(The app) should show the true image of us. But now as I look to this character, I see that we are not looking the same. They used the colour for white people.” [Female; IDI^b^ 4]	
	Targeting common problems	“I would like to get advice on things like sexual activities. The app would advise us on what will happen when we engage in sexual activities at an early age.” [P4; female; FG 5] “The app should also have pictures of the people smoking and what happens to them when they are smoking. It can also have pictures of the people who were addicted to alcohol and how they managed to stop drinking alcohol.” [P3; male; FG 2]	
**Confidentiality**			
	Creating a safe space	“(The app) will not judge you. It will be you and the app. Also confidentiality will be there. It won’t tell other people about your problems. That is nice idea.” [P5; female; FG 1] “Getting advice through an app would be a good idea because most of the time when you tell a person or a friend your problems, they don’t keep them to themselves; they tell people. If I share my problems on the app; I will get the advice that I need without worrying about what they will say about you to other people.” [P3; female; FG 5] “There will be a problem if [other people] can get to access what I am doing on the app.” [P4; male; FG 2]	
**Technological aspects**			
	Phone data	“I always run out of airtime to buy data and have to wait until month-end.” [Male; IDI 1]	
	Storage	“I’m always scared that [the phone] will run out of space.” [Female; IDI 3]	

^a^FG: focus group.

^b^IDI: in-depth interview.

#### Theme 1: App Features

Participants mentioned that the app should include stories of individuals who had been through difficult situations and narrate how they improved their circumstances. In addition, they suggested that the app should include points and difficulty levels to keep them motivated and engaged. In general, mobile games seemed to be very popular and were already used by many as a strategy to avoid rumination and exercise attentional control.

Combining both findings, the Kuamsha app was developed using a *narrative game format (app component 1)*. Under this approach, the app teaches the principles of BA through an engaging story. The stories were developed collaboratively with a storywriter (HC) using the findings from the conceptualization phase as the basis for the stories and drawing inspiration from other commercially available narrative smartphone apps (eg, “Episode”).

**Figure 2:**
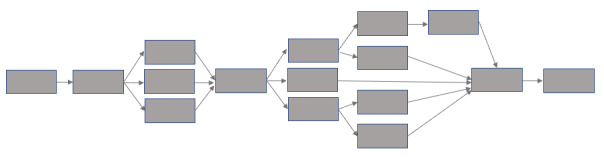
Branch-and-bottleneck game structure.

We developed 2 stories with the adolescents: the Song Contest and the Football Match. Each story details a challenge—to win a schoolwide song contest or play in a football match. Participants become a part of the story as one of the characters (which they are able to personalize). Each story consists of 6 modules (“episodes”) played in sequential order, and each module is designed to illustrate different BA skills (eg, self-care, problem-solving, and activity scheduling). To improve learning, participants can interactively choose actions that their story characters can undertake. These actions lead to different outcomes so that participants can understand the consequences of their actions. Participants have the opportunity to “correct” outcomes by reconsidering their actions. To keep the complexity manageable, story branches quickly merge with the main storyline through a branch-and-bottleneck structure (Figure 2). This approach allows the player to construct a relatively distinctive and personalized story while ensuring consistency across users [62]. A summary of each module is shown in Multimedia Appendix 2.

Learning is also facilitated by a story narrator that appears as a bird. As participants choose actions, the bird might “talk through” what the action would lead to or might sum up learning points as they occur in the story. To ensure that players reflect on the story and the concepts and choices made, each module ends with a summary of the lessons learned. This section includes a series of multiple-choice questions, which are saved as part of the game data for later analysis and potential review by peer mentors ahead of their weekly calls.

Each module was designed to take 15 to 20 minutes to complete, and adolescents were expected to complete at least 6 episodes from 1 story within an 11-week intervention period. We allowed users to progress freely within the app, permitting them to complete both stories if they desired to do so during the intervention period.

A key challenge in digital interventions is ensuring that the skills learned within the controlled environment of the app are effectively transferred to real-life situations. To enable this, the Kuamsha app integrates *real-life exercises (app component 2)* to ensure that the users put the BA skills learned into practice. As part of this component, participants are asked to think of a realistic and achievable goal to work on over the 11-week intervention period and schedule a series of weekly homework activities that align with each module.

Participants are asked to report how often they completed these homework activities and *monitor their mood (app component 3)* as they engaged in these activities. Users receive feedback on how their mood has progressed over time to highlight the relationship between what they do and how they feel. They are also reminded to report their progress on their weekly activities via *notifications (app component 4)*. Notifications are sent up to 4 times a week, but users are allowed to disable this function as some adolescents suggested that notifications were not always desirable.

Different *game design elements (app component 5)* were introduced, as suggested by the adolescents. First, the Kuamsha app includes character personalization. Second, it also includes in-app points that users can gain at different stages of the app journey (eg, every time they complete a module, report on their homework activities, and monitor their mood). Third, the Kuamsha app includes 2 different absorbing activities or “mini-games” designed to create immersive experiences to captivate players’ attention and foster a state of absorption [63]. One is a music absorbing activity, which consists of a rhythm game in which users tap the screen in time with the music. The other is a football absorbing activity, in which users practice taking shots on goal while avoiding distractors. Similar to other games that the adolescents mentioned playing in the qualitative work, we provided the option to adjust the difficulty level of these minigames as they progressed through the story.

#### Theme 2: Cultural Validity

Questions regarding cultural appropriateness emerged several times, along with the importance of incorporating elements specific to the study population. Adolescents wanted the story characters to look similar to them and the app to target common problems in their community. For example, many adolescents mentioned risky behaviors—such as alcohol, smoking *dagga* (cannabis), and teenage pregnancy—as important deterrents to achieving their goals. Although the app was not intended to target risky behaviors specifically, the stories were designed to cover topics such as drinking alcohol and relationships to make them more relatable to the target population. Special care was also taken to ensure that the stories were inclusive with regard to gender, age, appearance, and socioeconomic status.

#### Theme 3: Confidentiality

Another key theme that emerged was confidentiality. Adolescents saw the app as an opportunity to discuss their problems discreetly without feeling judged.

As many household members often share a single smartphone, numerous participants across both settings mentioned using security patterns and log-in codes to prevent unauthorized access to their personal content. This theme highlighted the need to *password protect (app component 6)* the Kuamsha app to create a safe space.

#### Theme 4: Technological Aspects

Within the technological aspects, adolescents discussed the lack of data and limited space as the 2 main barriers when using their smartphones. This theme prioritized a low-storage app and the need to explore features that would allow the user to access the app without internet connectivity.

Drawing from these findings, the Kuamsha app was designed to be accessible in both web-based and *offline modes (app component 7*). In this way, the app only requires an internet connection when it is accessed for the first time, during which the app automatically downloads the modules to the device’s internal storage. After that, the users can complete all modules at any other time regardless of the internet signal. Furthermore, the graphic elements were reduced to use little storage space and increase the app’s stability and performance.

### Prototyping Phase

Using the results from phase 1, a simple, scaled-down version of the app was developed (beta model). In total, 82 adolescents were involved in phase 2 and asked to provide feedback. A total of 3 qualitative research methods were used in this phase: usability testing sessions, focus group discussions, and participatory workshops.

During the usability testing sessions, adolescents were given smartphones and asked to interact with different apps (eg, Mood Tracker, Avatar Maker, and Magic Tiles). Each of these apps targeted a different app component (eg, mood monitoring, character personalization, and games that increased in difficulty). In addition, we designed different paper-based wireframes of other components, such as activity scheduling and allowing players to choose how the story unfolds. These sessions had two objectives: (1) to obtain feedback on the different app components and (2) to examine adolescents’ familiarity with smartphones. An excerpt of the results is presented in Textbox 1.

**Prototyping phase: excerpt of adolescents’ feedback on the app’s main components.**

**Personalization**

- “I enjoyed personalizing my character. I decided to make my character look that way because some of the features look like mine. The character looks almost like me even though I have also added the beard to show that one day when I grow up I would like to have a beard.” [Male; user testing session (UT) 4]
- “I liked it for the fact that I got to personalise my own character and I have added all the features that are similar to mine. I wanted the character to look like me.” [Female; UT 5]

**Mood monitoring**

- “I like the idea of rating myself and if I see that I need help, I can try new ways like going to the social workers.” [Male; UT 1]
- “I prefer to express my feelings through an emoji.” [Male; UT 3]
- “The emojis are very clear and they show clearly what they mean.” [Female; UT 5]

**Choosing how the story unfolds**

- “It is a good game to play. It shows you that before taking a decision between going to the tavern or preparing for the exam, you need to first think of the benefits that you will get out of each decision.” [P1; female; UT 7]
- “It would be fun for me because I would be getting to decide how the story goes until the end.” [P3; female; UT 8]

**Absorbing games**

- “I like this game a lot. It takes my concentration as by the time we met I was not happy but through it, now I’m starting to feel fine.” [Male; UT 1]
- “It is easy if you concentrate but hard to win. The more it becomes hard, the more you enjoy the game.” [Female; UT 2]
- “It was not difficult to play this game because you gave me instructions before I began playing, but I wouldn’t have found it easy to play it on my own.” [Male; UT 4]

Overall, adolescents were familiar with smartphones and enjoyed most of the app components, particularly those involving gamification. The subtheme of cultural validity re-emerged when participants were asked to personalize their characters. Adolescents preferred games that were challenging but, at the same time, easy to understand from the beginning. This feedback led to the development of an *onboarding process (app component 8)*, whereby the user would be introduced to the app and guided through its main components the first time they opened it. This onboarding process explains how to interact with the interface, choose their preferred language, and select one of the stories.

When asked to monitor their mood, adolescents preferred to use emojis to describe their feelings. An initial list of 15 emojis was created. Adolescents were shown these emojis without descriptions and asked to rate which emotions were represented to select the emojis that better captured the emotions portrayed in the mood monitoring (ie, unhappy and happy). Numbers in the slider were intentionally omitted to keep it simple, as suggested by the adolescents. To ensure the integration of the mood monitoring component into the game, we implemented a system in which users receive in-app points every time they complete mood monitoring and brief feedback on how their mood is progressing.

Focus group discussions were used to obtain feedback on the story characters, artwork, and background music. Adolescents were asked to list popular names in the community, and some of these names were used for the characters in the stories. In addition, the discussions served as a platform to address concerns about the limited access to mental health treatment in both study settings, which was a recurring theme raised by the participants. In response to these insights, we identified the need to design and integrate an *emergency button (app component 9)* to provide users with a direct avenue to seek immediate assistance during critical moments.

The participatory workshops reviewed and adapted the storylines and homework activities to ensure that they were appealing to adolescents in both study sites. As part of these workshops, adolescents were asked to read through the scripts and prepare a play in which they would role-play the stories. Following these discussions, modifications were made (in terms of language and content) to make the stories more locally and culturally relevant.

### Product Release Phase

The results from phases 1 and 2 provided input for developing an MVP of the Kuamsha app. The study team partnered with Sea Monster—one of Africa’s leading gaming companies—to develop the app.

The MVP was developed for low-cost Android Go devices, with testing taking place on Samsung Galaxy A2, the device used in the study. It was built on Unity (Unity Technologies), a cross-platform engine popular for mobile game development. The app has 2 critical dependencies. One is Ink, a plug-in that allows players to interact with the stories via multiple-choice answers or text fields. The other is Firebase, which captures player data on a web-based database and tracks users’ engagement with the app. The MVP was tested via usability testing sessions and a prepilot before rigorously evaluating it.

Usability testing sessions were conducted with 12 adolescents from Uganda and 12 from South Africa. Most adolescents (20/22, 91%) found the app a fun way to learn new skills and relax the mind. A field-worker observation in South Africa stated the following:

She looked very interested and engaged because she was smiling and giggling as she was reading the story.

Some differences were noted between female and male participants, with male participants preferring the “Football Match” story and female participants preferring the “Song Contest” story.

These sessions helped highlight some parts of the app that were not intuitive and needed refinement. For example, adolescents in Uganda struggled with literacy skills at a greater level than anticipated. According to census data from the Wakiso District, 85% of adolescents in the study site area are literate [53]. However, although many adolescents could decode app content, some had difficulties with reading comprehension. Owing to time and resource limitations, it was decided that only adolescents with an acceptable reading comprehension level would be enrolled in the feasibility study in Uganda. The text’s complexity was assessed using a web-based tool that corresponded to a primary 7 reading level. Eligible participants were required to score ≥83% on a reading comprehension assessment drawn from the Young Lives study [64].

Similarly, when asked to choose a goal, most adolescents set goals related to their lives, but these were rarely specific, measurable, or time based (eg, *“*I want to make wise decisions” or “I want to work hard”). This highlighted the critical role of peer mentors in making sure that goals were meaningful and achievable within the 11-week intervention period. On the basis of these findings, we adjusted the app so that the goals and homework activities could be modified anytime the user wanted.

A prepilot study was conducted in the South African study site with 17 adolescents who screened positive for mild to moderately severe depression based on scoring between 5 and 19 on the 9-item Patient Health Questionnaire modified for adolescents (PHQ-A). The adolescents were asked to use the Kuamsha app for 11 weeks. They were allocated to a trained peer mentor from whom they received 6 weekly phone calls. App engagement metrics were collected throughout this time. Table 3 shows the sociodemographic characteristics of study participants at baseline.

**Table 3:** Prepilot study: sociodemographic characteristics of study participants at baseline (N=17).

Variables	Values
Female participants, n (%)	15 (88)
Age (y), mean (SD)	16.2 (1.07)
School grade, mean (SD)	10.4 (0.80)
Ever repeated a grade, n (%)	4 (24)
Has children, n (%)	2 (12)
Lost a parent, n (%)	3 (18)
Ever used a smartphone before, n (%)	14 (82)
Did any work for pay, n (%)	0 (0)
Simple Poverty Scorecard^a^, mean (SD)	43.5 (16.14)

^a^The Simple Poverty Scorecard total score ranges from 0 (most likely below a poverty line) to 100 (least likely below a poverty line). An average score of 43.5 on the Simple Poverty Scorecard corresponds to a poverty rate of 57.3%. This was computed using the South African upper national poverty line, which is set at 32.57 South African rands per person per day (£2 in 2017 prices [US $2.58]) [65].

The average PHQ-A score was 7.2 (range 5-15). In total, 12% (2/17) of the participants had moderate levels of depressive symptoms (score between 10 and 19), and the rest (15/17, 88%) were mildly depressed (score between 5 and 9). A total of 41% (7/17) of the participants endorsed the ninth item on suicidal ideation. These participants were referred to the trial psychologist to assess whether they needed any additional intervention. After the assessment, the trial psychologist verified that none of these adolescents had identified means or plans to attempt suicide, and as such, they were included in the prepilot.

Table 4 shows the app engagement metrics for 94% (16/17) of the participants (there was a problem linking the device with the game data for 1/17, 6% of the participants). On average, the Kuamsha app was launched 38.4 times per participant, and users spent approximately 4 hours on it over the intervention period. Most participants (13/16, 81%) opened both stories, and most (12/16, 75%) completed at least 6 modules (the recommended number of modules to be completed during the intervention). The average number of modules completed was 8.8, and several adolescents (7/16, 44%) completed both stories (12 modules in total). Although, on average, this reflects strong engagement with the app, it is worth highlighting that 25% (4/16) of the participants did not complete the recommended modules. Among this group, 6% (1/16) of the participants only completed 2 modules and 19% (3/16) completed 4 modules. Importantly, there was no statistically significant difference in sociodemographic characteristics between participants with lower and higher engagement. We also found no differences in depressive symptoms during screening or at endline.

**Table 4:** Kuamsha app engagement metrics in the prepilot study (N=16).

	Values
Number of log-ins, mean (SD)	38.4 (23.6)
Time spent on the app, mean (SD)	4 h, 13 min (2 h, 40 min)
Opened both stories, n (%)	13 (81)
Completed at least 6 modules, n (%)	12 (75)
Number of modules completed, mean (SD)	8.8 (3.7)
Number of set-up weekly activities, mean (SD)	8.3 (4.0)
Number of completed weekly activities, mean (SD)	47.2 (34.7)

To further explore the factors influencing varying levels of engagement, we plan to conduct in-depth interviews during the feasibility studies in Uganda and South Africa and stratify participants based on high versus low app engagement. These qualitative data will help guide improvements in future iterations of the app.

Adolescents’ goals during the intervention period included improving communication with friends or family, making new friends, studying more often, and exercising more. On average, participants set-up 8.3 weekly activities and recorded completing them 47.2 times. In total, 25% (4/16) of the participants used the emergency button. Of these 4 participants, 3 (75%) made a single call using the button, whereas 1 (25%) used it on 3 separate occasions. The peer mentors had access to these data ahead of every weekly call, and they were trained to follow up on such instances. Example screenshots of the app and its main components are shown in Multimedia Appendix 3.

## Discussion

### Principal Findings

This study described the user-centered development of a smartphone app to reduce depression among adolescents in a sub-Saharan African context and summarized the findings of multicycle usability testing.

The smartphone app, called the Kuamsha app, is a gamified app that uses storytelling techniques and game design elements to deliver BA therapy in an engaging way. Central to the study was an iterative co-design process with adolescents from rural South Africa and Uganda. Extensive formative research was conducted with 160 adolescents, who guided the development and provided feedback at each developmental phase.

The user-centered development was carried out in 4 phases, each combining different research methods and elicitation techniques to ensure that a depth and breadth of user perspectives was incorporated into the design. In addition, a broad array of local stakeholders across both study settings was consulted during the development process to ensure that the intervention was culturally acceptable within communities. This iterative methodology enhanced the app’s acceptability, cultural relevance, usability, and validity with the targeted population.

Adolescents’ preferences regarding a mental health app revolved around 4 main themes: app features, cultural validity, confidentiality, and technological aspects. These themes informed the specific components of the app, which included (1) a narrative game format that leverages the power of storytelling and immersive narratives to create a dynamic and engaging experience for users, (2) integration of real-life exercises and homework activities within the app to ensure that the users practiced the BA skills learned in the app, (3) mood tracking to help adolescents recognize the link between mood and behavior, (4) push notifications to remind users to report their progress on their homework activities, (5) game design elements (character personalization, in-app point system, and absorbing “mini-games”), (6) password-protected access for security, (7) an offline mode allowing users to play on the app without an internet signal, (8) an onboarding process to guide users through the app, and (9) an emergency button to refer adolescents at risk of suicide.

The results of multicycle usability testing sessions showed very high engagement metrics. Some of our engagement metrics were significantly higher than those of other apps that have been reported in the literature [24,66,67]. These preliminary results suggest that the Kuamsha app is acceptable in terms of usability and engagement. In total, 2 studies are currently underway to rigorously test the intervention’s feasibility, acceptability, and efficacy in reducing depressive symptoms.

Overall, this study contributes to the broader literature on digital mental health interventions, particularly regarding the importance of involving potential users and key stakeholders as active collaborators in intervention design [57,68]. It also highlights the potential of leveraging storytelling and games as an effective strategy to maintain user engagement and enhance learning [69]. Finally, and in line with previous findings, our results show the importance of developing an app that is relatable and caters to the unique needs, challenges, and preferences of the target user [68]. Given the limited number of apps specifically developed for (and with) adolescents in sub-Saharan Africa, this study fills a critical gap and serves as a pioneering effort to create a culturally relevant and acceptable intervention for addressing depression among this population.

### Limitations

This study has several limitations. First, a convenience sampling strategy was used to recruit adolescents from both study sites, which may have resulted in some selection bias. However, the relatively large number of adolescents consulted may have helped minimize this. Second, most of the formative work was conducted with adolescents from the general population and not specifically with adolescents identified as having depression. The exception to this is the prepilot study, which included adolescents who screened positive for mild to moderately severe depression on the PHQ-A. Although feelings of hopelessness and self-harm did arise during the discussions (particularly during one-to-one sessions), more formative work with adolescents with depression might have helped ensure that the app was optimized to address their needs and concerns. However, the app was thoughtfully designed with several features aimed at supporting user engagement. First, the app was designed to be supported with weekly phone calls from peer mentors, serving to sustain user engagement and address issues related to low motivation. Second, the app includes reminders to help users stay on track and overcome challenges related to memory and concentration frequently associated with depression. Third, recognizing the nonlinear nature of depression recovery, the app allows users to navigate setbacks and relapses without feeling discouraged. The results of the DoBAt Study will provide more information about the feasibility and acceptability of the Kuamsha app among adolescents with depression [59]. Fourth, although this study was conducted in 2 diverse sub-Saharan African settings, contextual differences should be taken into account when considering the transferability of the study findings to other sub-Saharan African populations. Fifth, the formative work revealed literacy difficulties among adolescents in Uganda, and therefore, a reading comprehension test was included as part of the screening criteria in the feasibility study in Uganda, which might have excluded the most vulnerable adolescents. Further app development work should explore the use of audio voice-overs or alternate features to increase the accessibility of the app to low-literacy populations.

### Conclusions

Although there has been a significant increase in digital mental health interventions in recent years, many of these platforms suffer from low uptake, slow rollout, and unsustainable business models. Furthermore, few interventions have been rigorously designed and evaluated for adolescents in sub-Saharan Africa or are available in local languages. Given the limited access to effective interventions in LMICs, innovative, scalable treatment delivery options are urgently needed.

Benefiting from the rapidly growing penetration of mobile devices, the Kuamsha app could help bridge the large treatment gap in South Africa, Uganda, and potentially other similar settings. If the feasibility studies produce promising results, they will inform the development of a further larger randomized controlled trial to support better management of depression among adolescents in low-resource settings while simultaneously strengthening primary and community-based health care systems.

## Appendix

Multimedia Appendix 1 Samples of interview guides and elicitation techniques.

Multimedia Appendix 2 Description of storylines.

Multimedia Appendix 3 Kuamsha app screenshots.

## Abbreviations

BA: behavioral activation
ELA: Empowerment and Livelihood for Adolescents
LMIC: low- and middle-income country
MVP: minimum viable product
PHQ-A: Patient Health Questionnaire modified for adolescents
Wits: University of the Witwatersrand

## Data Availability

The participant information sheet provided to study participants did not include information regarding data sharing. Consequently, data from this study, which include transcripts and audio files, will not be made available to the public. Data supporting the study findings can be found in the translated quotations in the *Results* section.
