# Supplementary material for: User-Centered Design of a Gamified Mental Health App for Adolescents in Sub-Saharan Africa: Multicycle Usability Testing Study
Source: JMIR Form Res. 2023 Nov 30;7:e51423. doi: 10.2196/51423 (PMC10722378; doi:10.2196/51423)
Supplement: Multimedia Appendix 1 [file formative_v7i1e51423_app1.docx]

Appendix 1. Samples of interview guides and elicitation techniques

| **Phase** |  |
| --- | --- |
| **(1)**  **CONCEPTUALISATION** | - People have different​ dreams. ​What do people your age in this community dream for their future? - Many teenagers feel sad or stressed at times. What words do people in this community use to talk about feelings ​depressed/low/down​? - We are going to ask you about a time when you set a goal that is important to you and worked towards it. This can be any goal, small or big. What was the goal you set? Why did you decide this goal was important to you? Did you achieve your goal? - Describe an average day in your life with your phone. - What sort of things do adolescents like yourself like/dislike doing on their phone? - Imagine that if you were feeling sad and that there was a ​mobile phone​ ​app​ that could give you support and advice and connect you anonymously with people that could help. How would you feel about such an app? PROMPT: Would you rather speak to a friend in person about your problems? If you like the idea, what is appealing about it? What sort of things would the app need to have to make it useful (these can be photos, videos, music, etc.)? What would make you stop using the app? |
| **(2)**  **PROTOTYPING** | *Paper-based wireframes designed to test different components of the app:*   - Activity scheduling   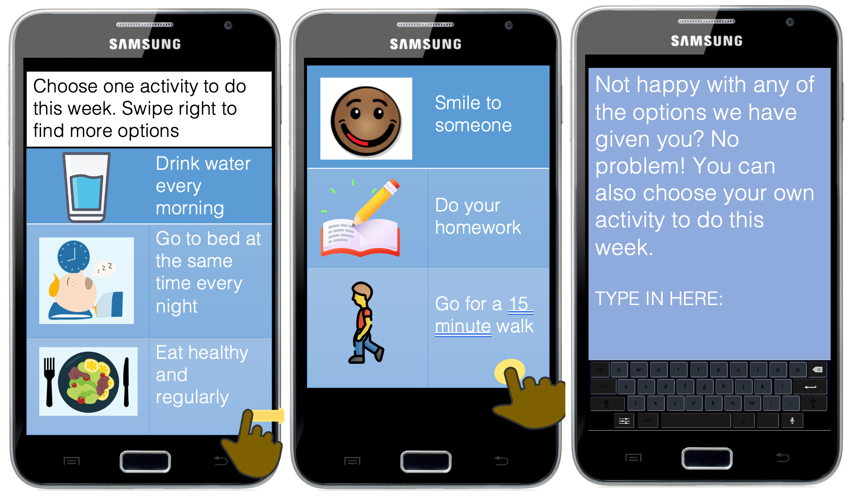   - Getting advice through the app   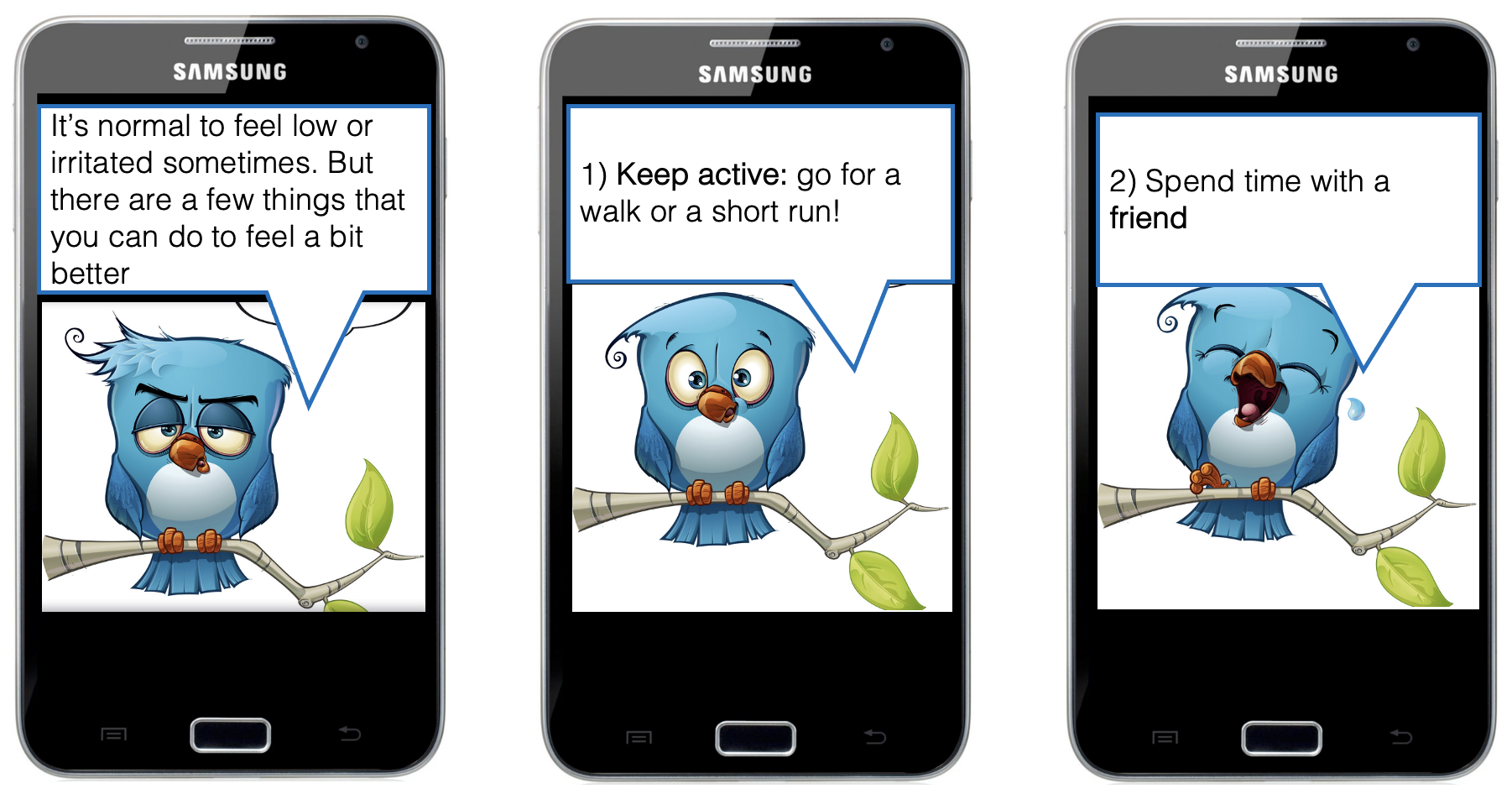   - Allowing players to choose how the story unfolds   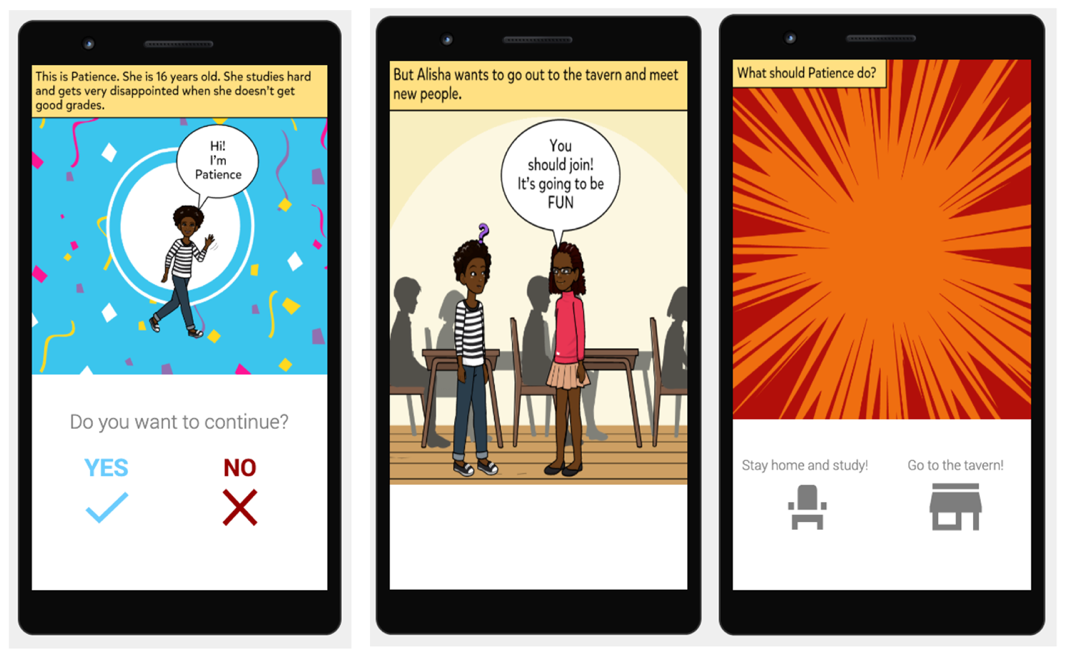 |
|  | *Participatory workshops – interview guides*   - What did you think about the story? What did you like or not like about this story? - Can you summarize the story? - What did you think about the characters? - What did you think about the setting/ place where the story happened? - What did you think about the way this story was written and the language that was used? |
| *Example mock-ups of the first basic prototype of the Kuamsha app* | |

| *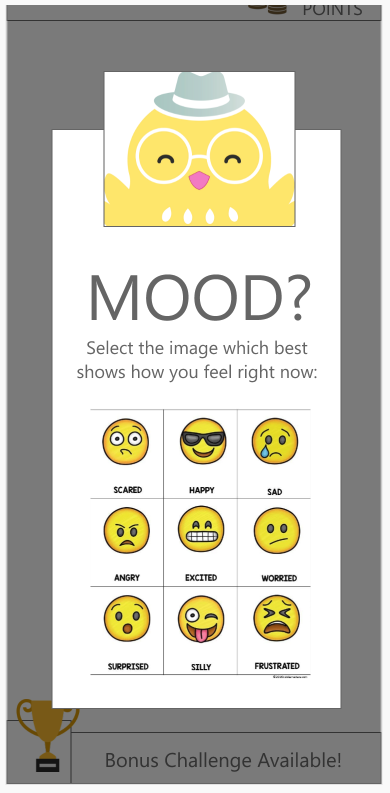* | *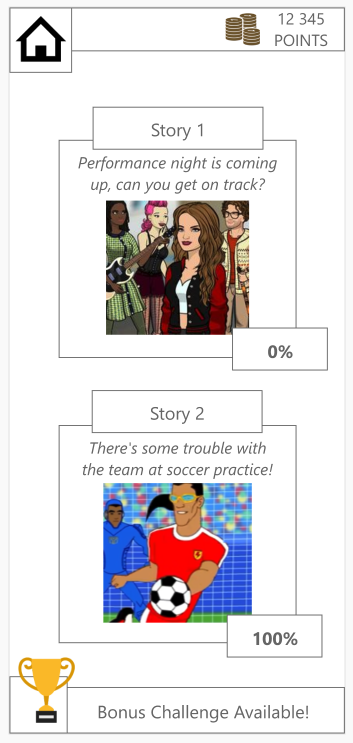* | *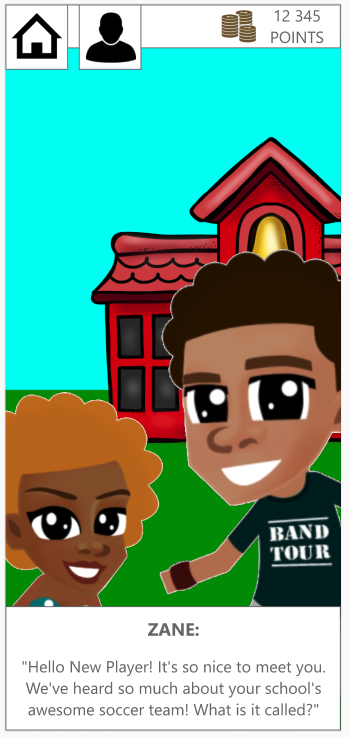* |
| --- | --- | --- |
| *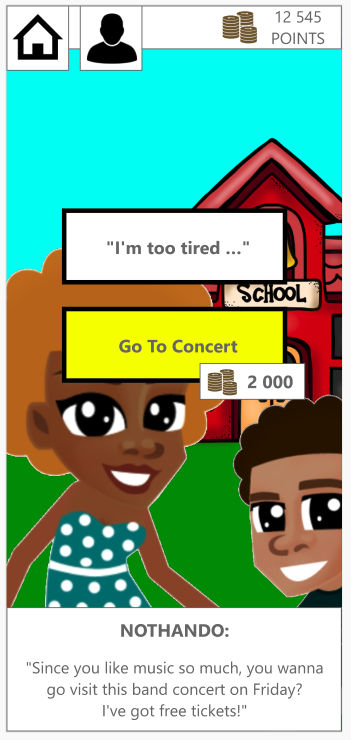* | *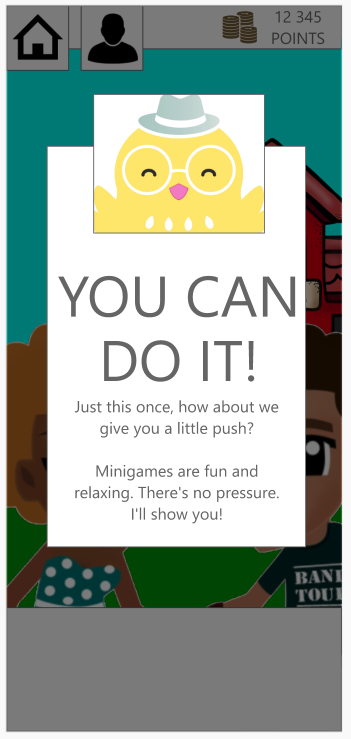* | *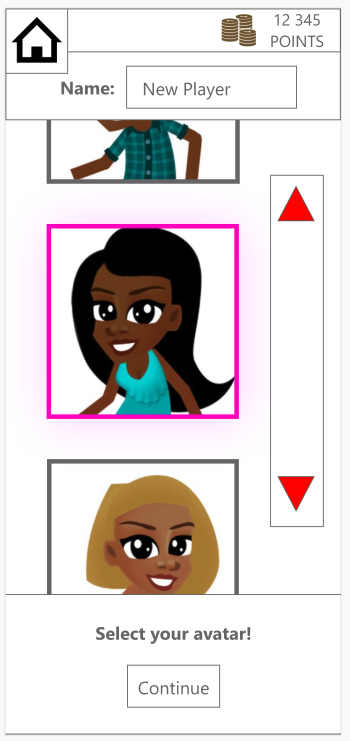* |
