# Supplementary material for: User-Centered Design of a Gamified Mental Health App for Adolescents in Sub-Saharan Africa: Multicycle Usability Testing Study
Source: JMIR Form Res. 2023 Nov 30;7:e51423. doi: 10.2196/51423 (PMC10722378; doi:10.2196/51423)
Supplement: Multimedia Appendix 2 [file formative_v7i1e51423_app2.docx]

Appendix 2. Description of storylines

*The Song Contest Story*

| **Learning module** | **Description of each module** | **BA Learning principles** |
| --- | --- | --- |
| **Episode 1**  **(“Pick a team”)** | Students find out about a school Song Contest. The winning prize is a voucher to shop for a new outfit at the mall. User chooses the main character’s name and picks two teammates to join the Song Contest.  Main character gets anxious about the idea of performing in front of everyone else. Christine (teacher) talks about the benefits of stepping outside one’s comfort zone and offers her support.  At the end of the module, the user will be asked to set a goal to work on over the 10 weeks of intervention. | Absorption  TRAP-TRAC  Sleep  Self-confidence  Relapse prevention |
| **Episode 2**  **(“Find a cool tune”)** | First practice session with group which didn’t go well because one teammate fell asleep. Main character is frustrated and decides to ask Christine for advice. After hearing that teammate’s grandmother ill, main character showed leadership and compassion in how they handled the situation. |  |
| **Episode 3**  **(“The Lure”)** | The team worked well together and made progress on the song. At the end of the practice, Prince (desirable character) invites the main character to go to the tavern. Main character is asked to think about the consequences of their actions but decides to go anyway. |  |
| **Episode 4**  **(“The Fallout”)** | The next day the main character is exhausted, hungover, and unable to concentrate. They arrive late to practice session without homework done. Teammates get annoyed. Main character decides to apologise instead of avoiding the problem and their teammates are forgiving. Main character learns about the importance of sleep and goes to sleep early. |  |
| **Episode 5**  **(“The Return”)** | Main character sleeps and feels recharged. Team is happy with the practice. Song is completed. Main character learns that they have to give a presentation if they win and asks Christine for advice. Main character learns ways to deal with being nervous and how to feel more self-confident. |  |
| **Episode 6**  **(“The Big Day”)** | Team feels nervous before the show. They perform and win the prize. Group reflects on journey. |  |

*The Football Match story*

| **Learning module** | **Description of each module** | **BA Learning principles** |
| --- | --- | --- |
| **Episode 1**  **(“Pick a team”)** | Main character is the striker for the local team. User chooses the main character’s name and picks a name for the football team. User gets introduced to the football task. At the end of the module, the user will be asked to set a goal to work on over the 10 weeks of intervention. | Absorption  Rumination  TRAP-TRAC  Problem-solving  Negotiation |
| **Episode 2**  **(“Game Over”)** | Main character misses some important shots. Shane (antagonistic character) is a discouraging main character because of their poor performance. Team loses the game. Main character leaves the match alone feeling upset and discouraged, ruminating over her performance during the match. Bird offers different, more positive perspective on performance but the main character not in a frame of mind to believe it. |  |
| **Episode 3**  **(“Hide away”)** | Main character is frustrated, misses school and gets a detention. Main character feels a bit better after friends visit her/him at her house, but apprehensive about going to school. Main character plays the football task which makes them feel energised enough to do their homework. |  |
| **Episode 4**  **(“That’s okay”)** | Main character returns to school feeling more positive after a good night’s sleep. Main character handles Shane well, but their mood takes a dip. Main character decides to speak with Coach Bayer after school about their performance in the match. Main character learns that mistakes can be useful opportunities to learn, instead of something to avoid. Main character practises what they’ve learnt and starts to feel more confident**.** |  |
| **Episode 5**  **(“Keep practising”)** | Main character speaks with friends before practice about what they learnt from Coach Bayer and asks for help in dealing with Shane. With their supportive presence, main character gets Shane to agree to stay away from them so that they can focus on football and winning.  Main character feels confident, keeps practising, and feels ready for the re-match. |  |
| **Episode 6**  **(“The Big Match”)** | It all came together for the main character, stepping out onto the pitch with new confidence. Main character keeps calm and meets their mistakes with curiosity instead of frustration. Main character scores the winning goal. |  |
