# Supplementary material for: User-Centered Design of a Gamified Mental Health App for Adolescents in Sub-Saharan Africa: Multicycle Usability Testing Study
Source: JMIR Form Res. 2023 Nov 30;7:e51423. doi: 10.2196/51423 (PMC10722378; doi:10.2196/51423)
Supplement: Multimedia Appendix 3 [file formative_v7i1e51423_app3.docx]

**Appendix 3. Kuamsha app’s main components and screenshots**

| **App component** | **Description** | | **Screenshot** |
| --- | --- | --- | --- |
| Log-in unlock code (app component 6) | Kuamsha is password-protected. Users need to enter a password every time they access the app. | | 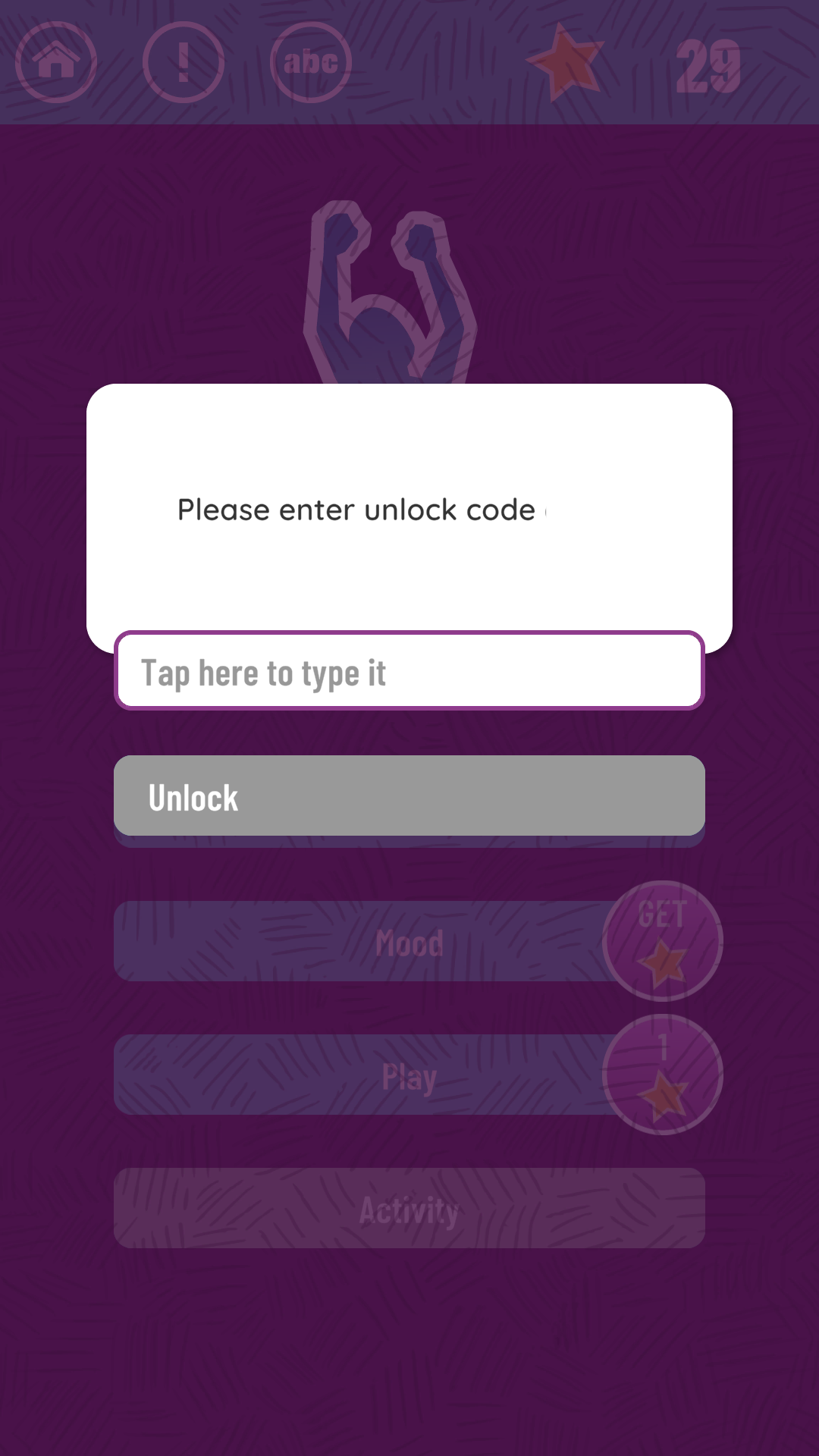 |
| Home screen | This is the first screen that users see as they log in to the Kuamsha app. Users have the option to play through the stories, monitor their mood, play absorbing activities to improve focus, or report on their weekly activities (see the rest of the table for further details on each of these components). | | 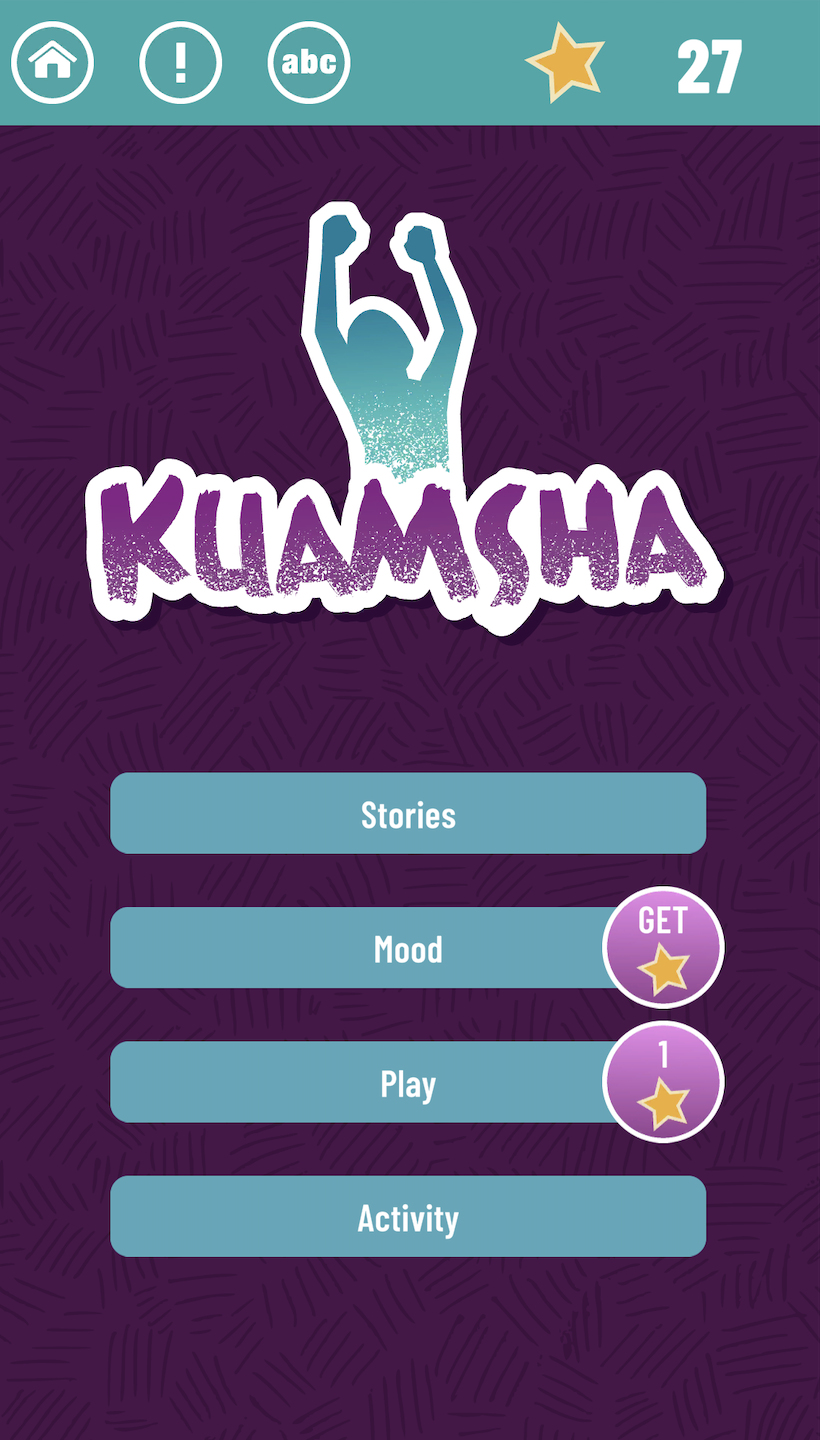 |
| Onboarding process (app component 8) | When users open the app for the first time, they are introduced and guided through its main components. This onboarding process aims to teach adolescents how to interact with the interface, choose their preferred language, locate the emergency button, and select one of the stories. | | 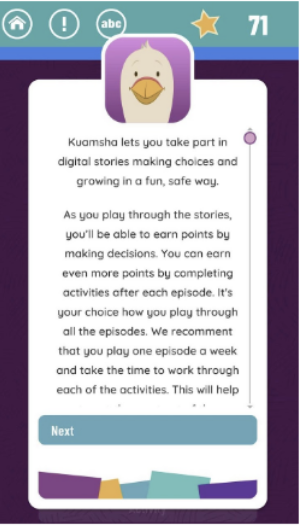 |
| Language selector | Users can select their preferred language. All the text on the app underwent 2 rounds of translation and has been checked by a clinical psychologist for accuracy. | | 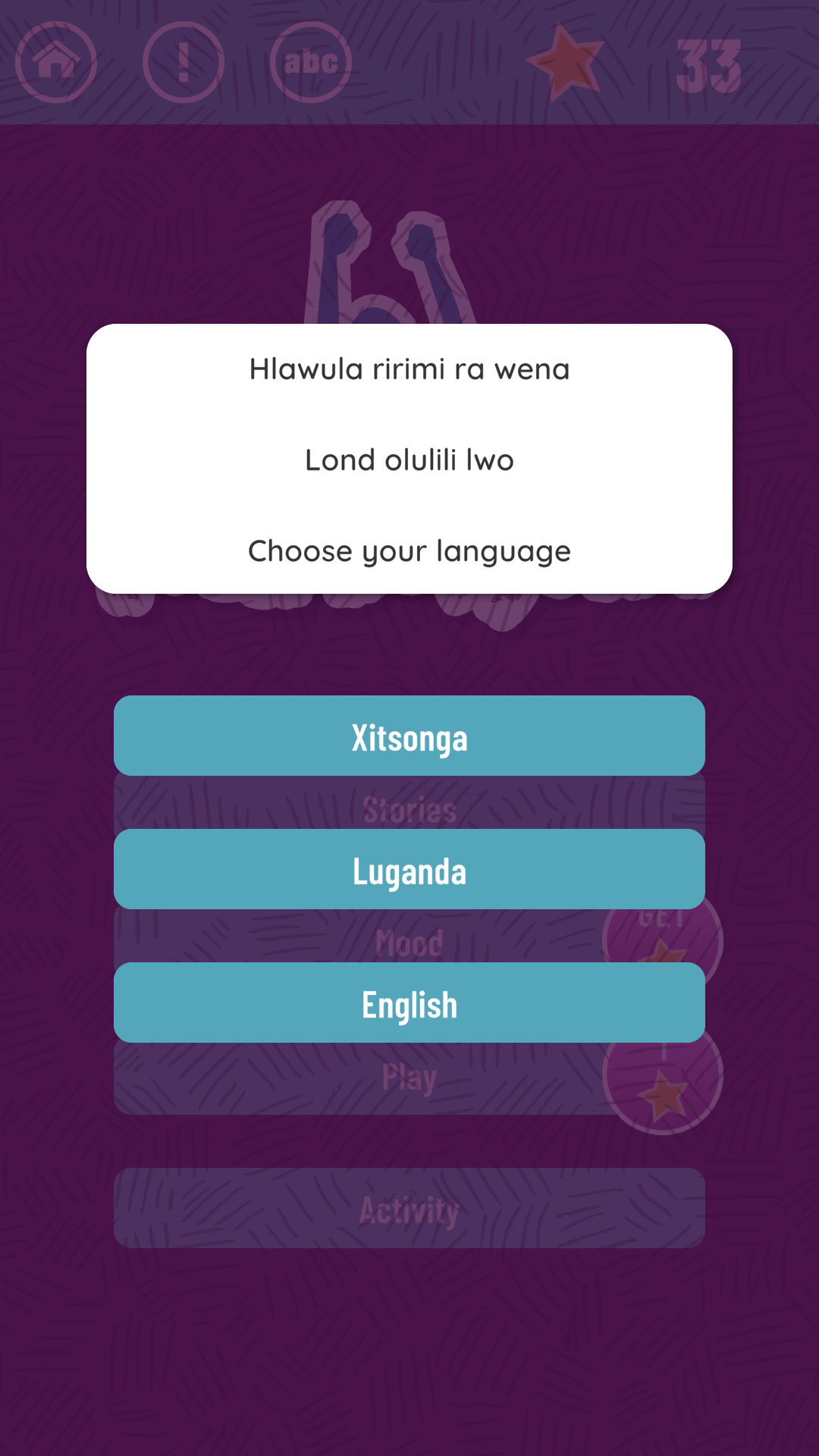 |
| Story selection (app component 1) | The core of the game consists of a choice between 2 narrative stories. Each consists of 6 modules that are played in sequential order. It is possible to begin one story and then switch to the other. During gameplay, points are earned for the choices made and by completing other core game elements described in this table. | | 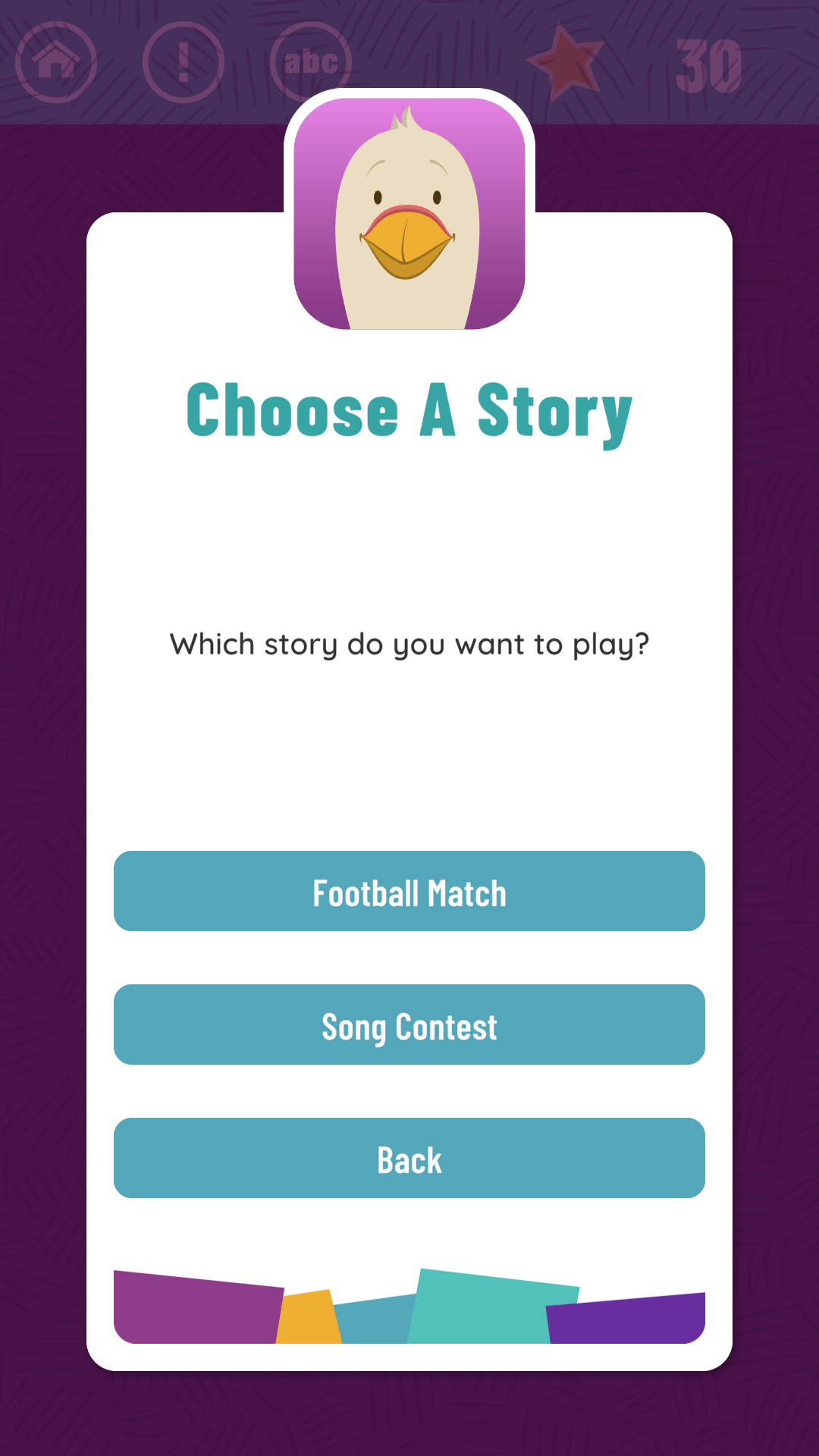 |
| Choices affect outcomes (app component 1) | A key principle of the game is that the player is drawn into the narrative through the use of decision points that allow users to shape the narrative. Choices made by the player branch the story in ways that lead the character down different pathways in the game. | | 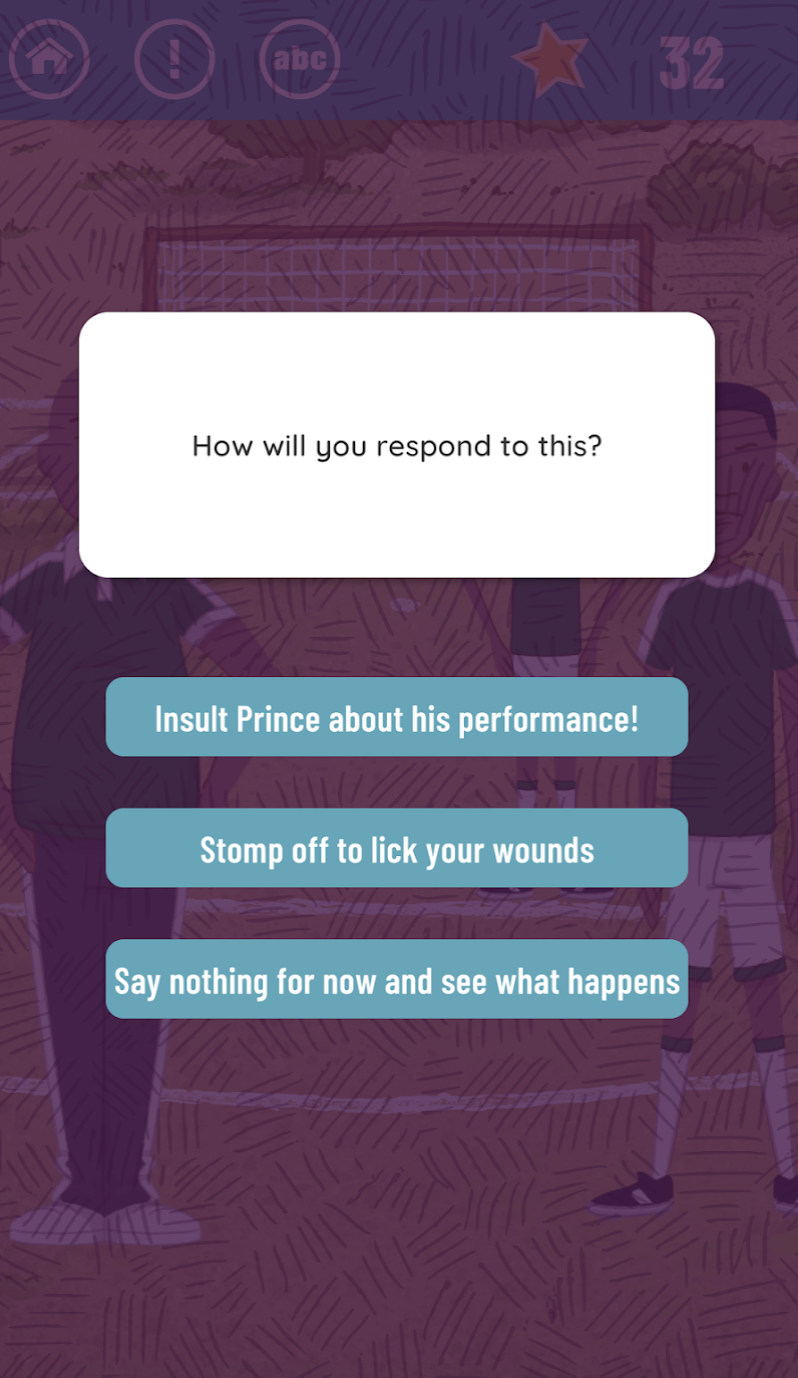 |
| Bird guide (app component 1) | The bird character is used throughout the game as a way of having players reflect on their choices, supporting review of what has been learned and offering suggestions and other game mechanics designed to draw the multiple components of the game into a unified whole. | | 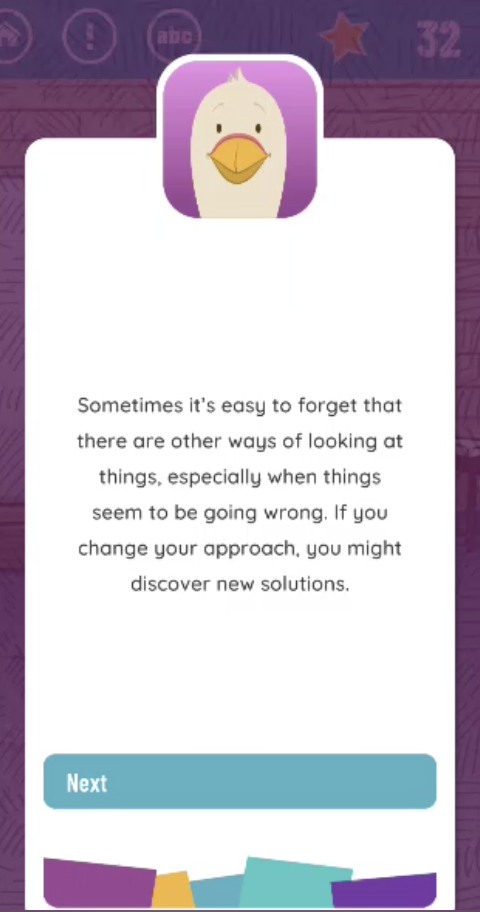 |
| Summary of lessons learned (app component 1) | To ensure that players are reflecting on the game and show some level of understanding of the concepts and choices made, each story episode ends with a summary of lessons learned. | | 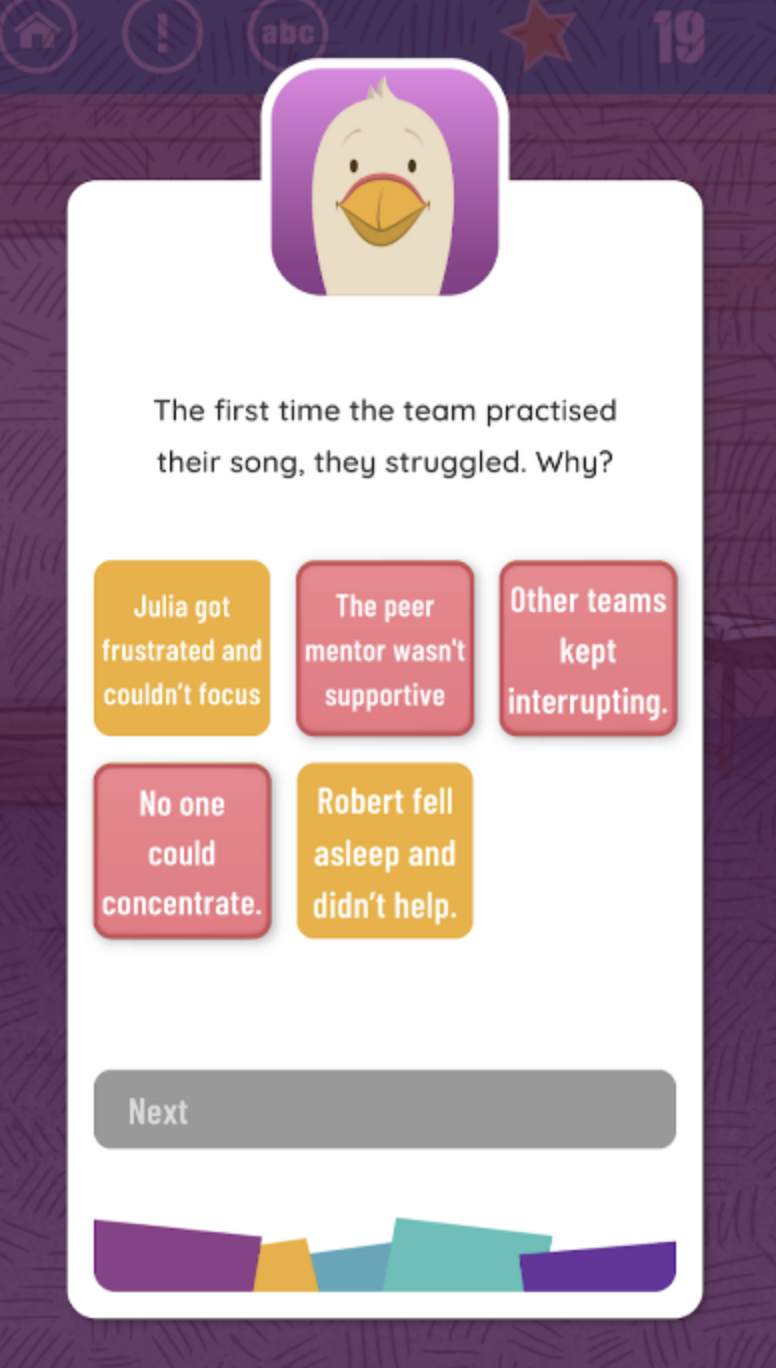 |
| Real-life exercises (app component 2) | Users are asked to think about an activity that they could do that is associated with the principle of the episode they just completed. Each homework session consists of 1 or 2 activities that expand on the behavioral activation principle covered in that session. | | 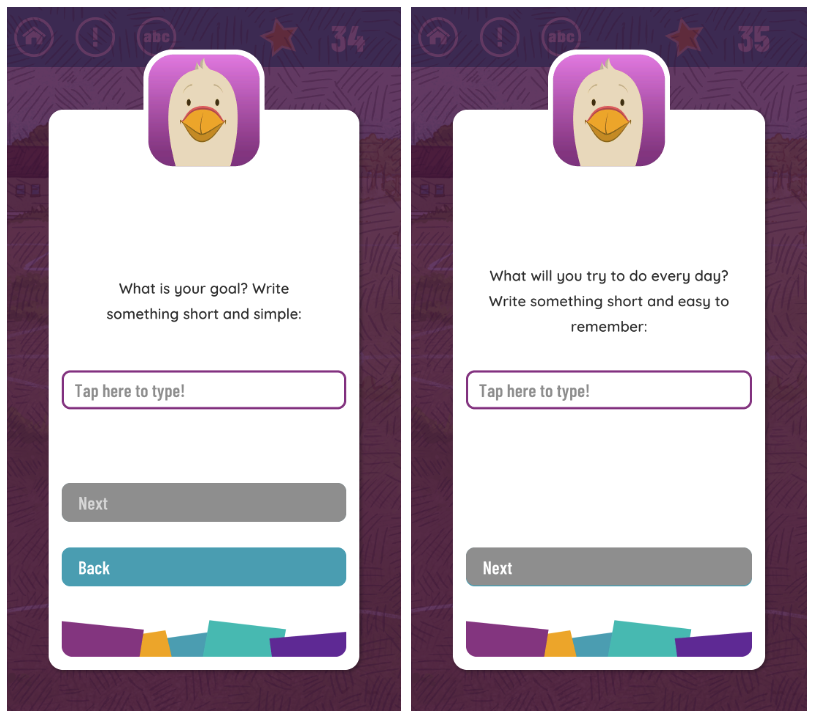 |
| Mood monitoring (app component 3) | Participants are asked to monitor their mood at different times (before and after the episode, when they report on homework, and when they complete an absorbing activity). Users receive feedback on how their mood changes over time and in-app points every time they complete the mood monitoring. | | 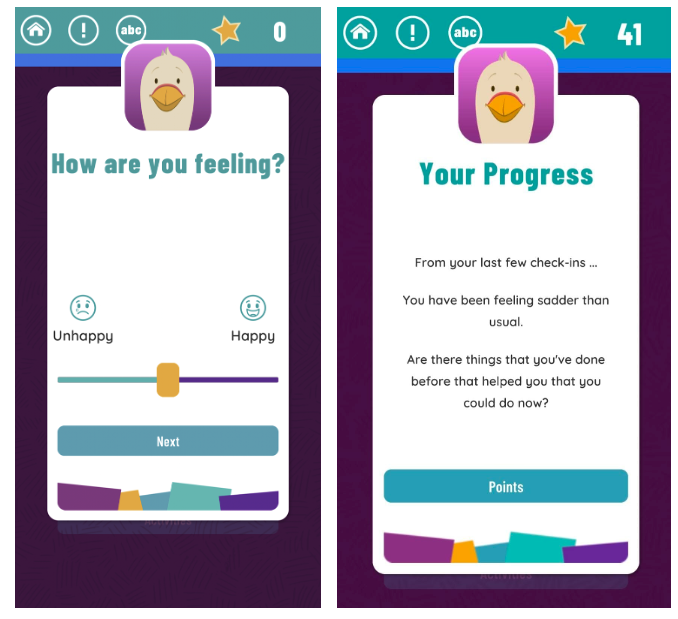 |
| Notifications (app component 4) | Users are reminded to report their progress on their weekly activities via notifications. | | 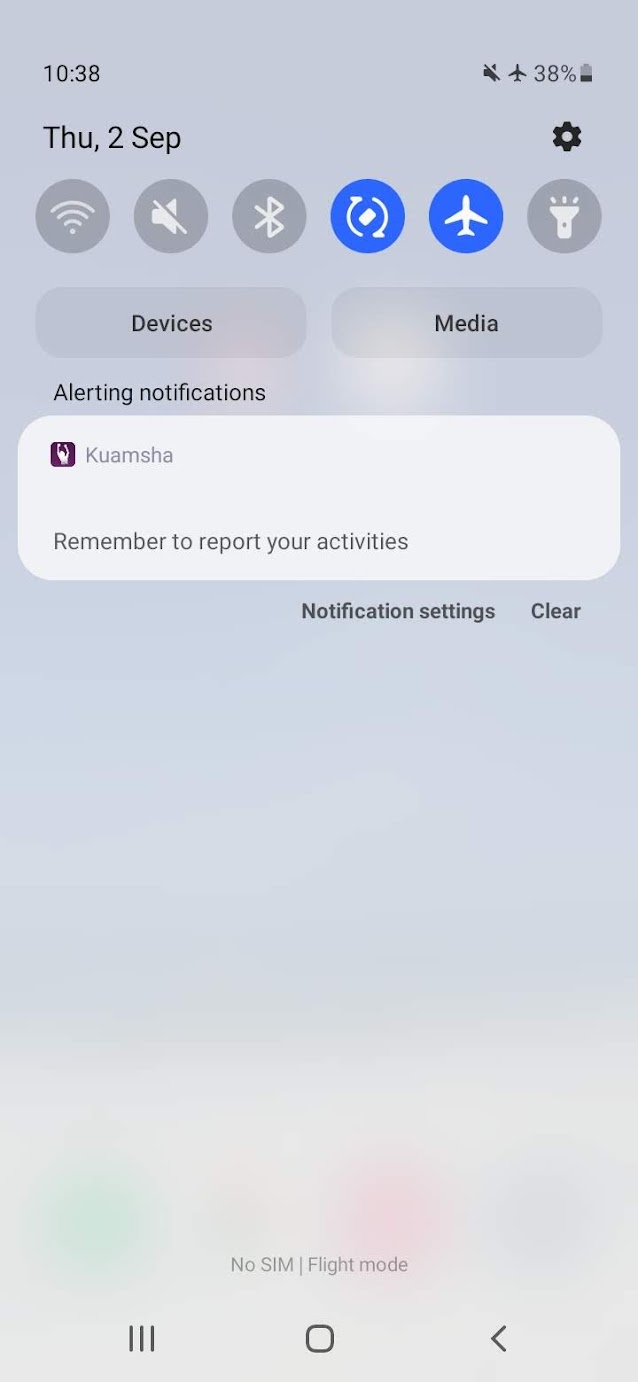 |
| Game design elements: personalization (app component 5) | Users are asked to personalize their character in the story by choosing their name, their preferred pronouns, and their team’s name. | | 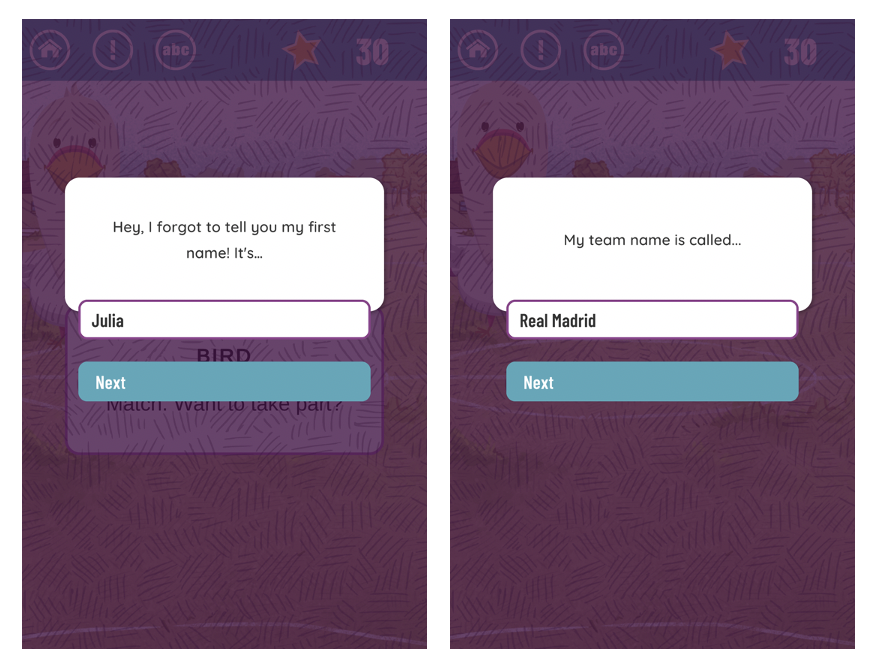 |
| Game design elements: in-app points (app component 5) | Participants earn in-app points every time they complete an episode, report their weekly activity, monitor their mood, and play the absorbing activities. | | 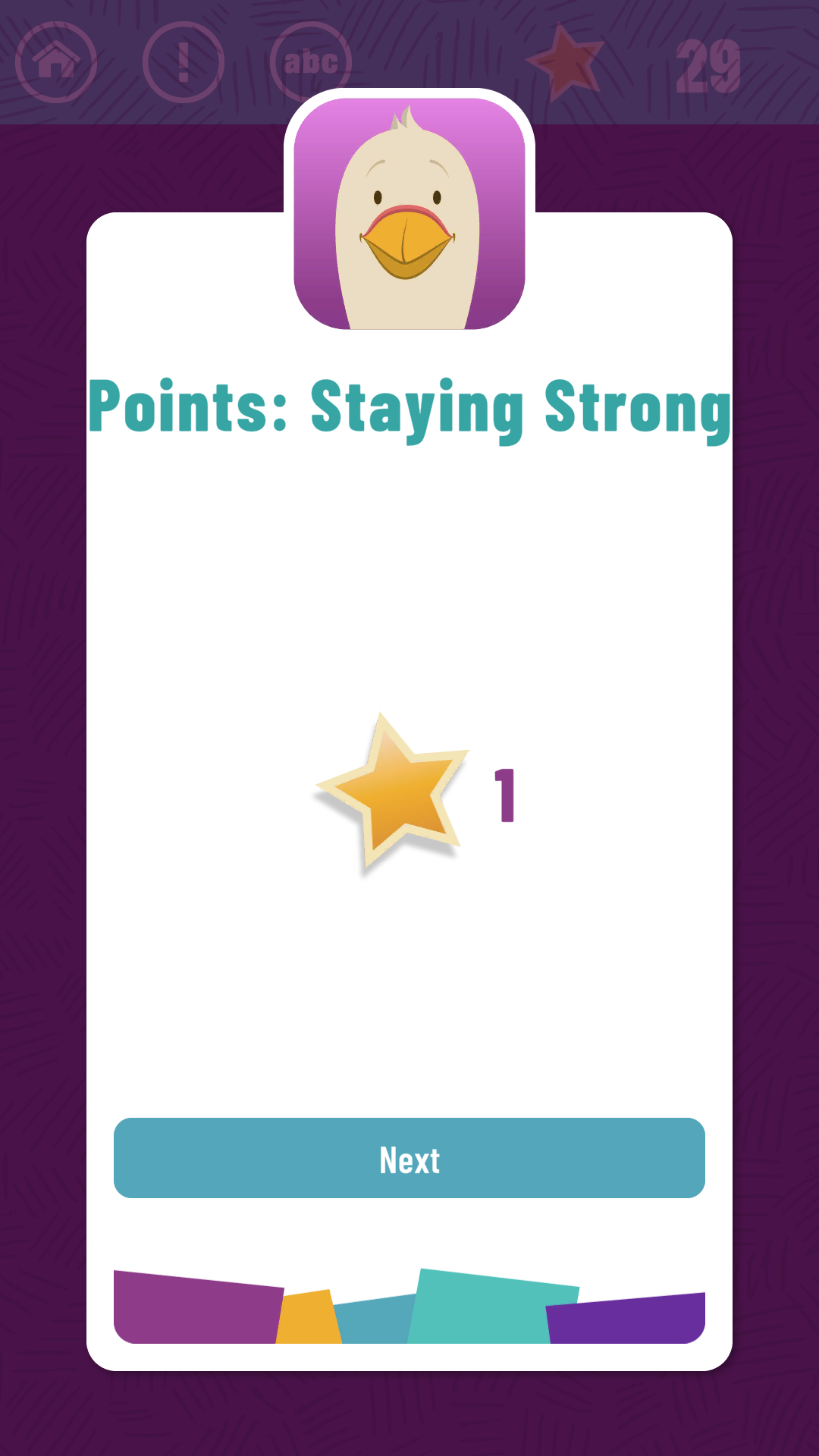 |
| Game design elements: absorbing activities (app component 5) | Kuamsha includes 2 different activities to teach the concept of absorption (focusing on an activity to detract from negative thoughts). Participants can decide between 2 different absorbing activities: a music absorbing activity (rhythm game in which users tap the screen in time with the music) and a football absorbing activity (users practice taking shots on goal). | | 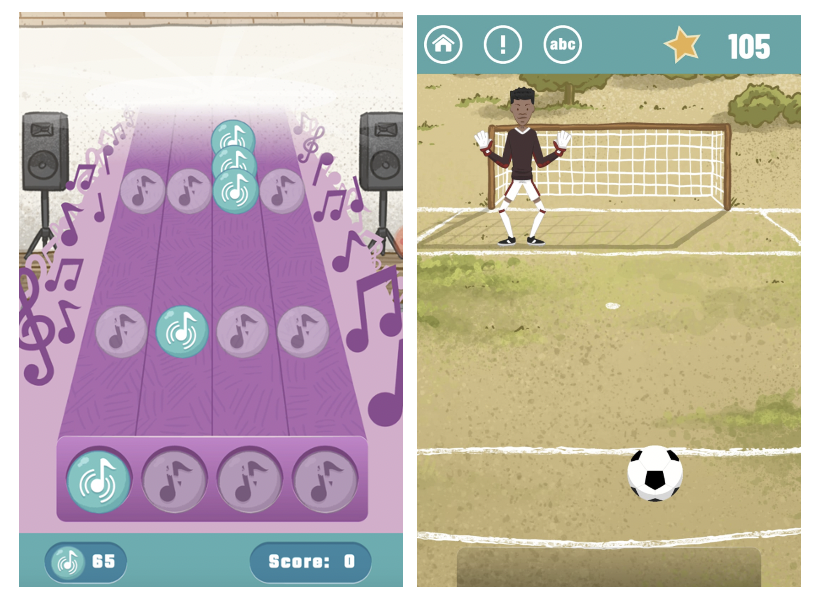 |
| Emergency button (app component 9) | Kuamsha includes an “emergency button” for adolescents to call in the event that they are feeling extremely sad or thinking about hurting themselves. | | 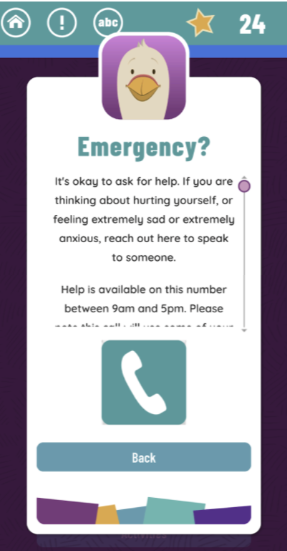 |
| Story characters | | 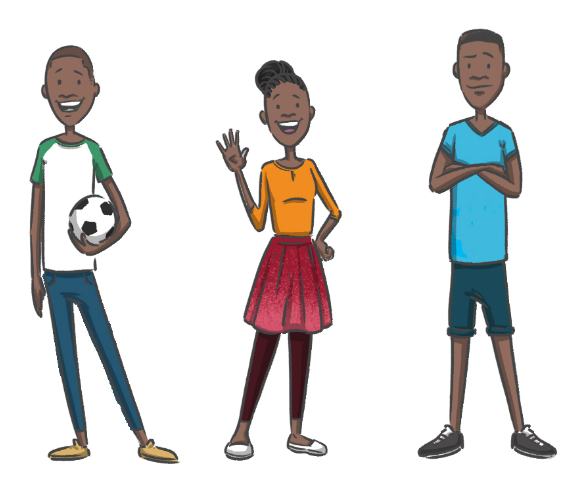 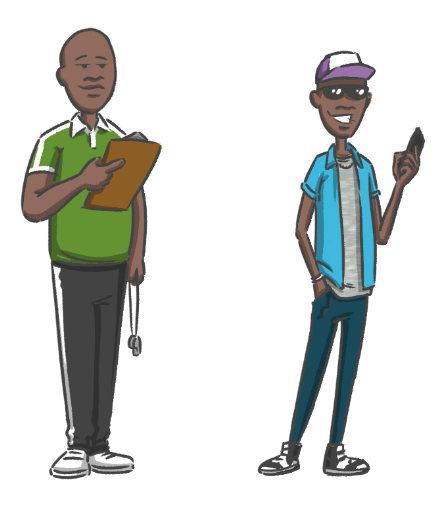 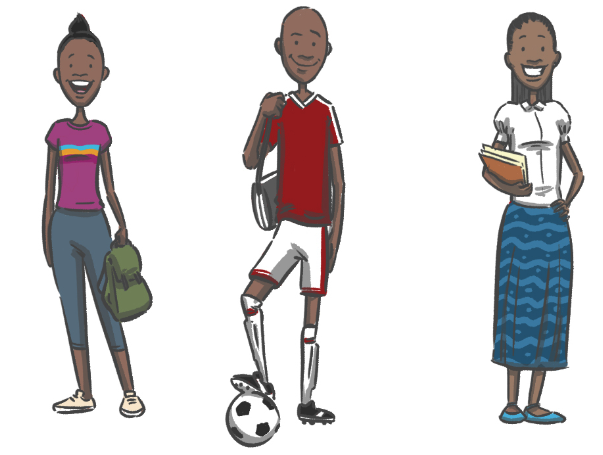 | |
| Background | | 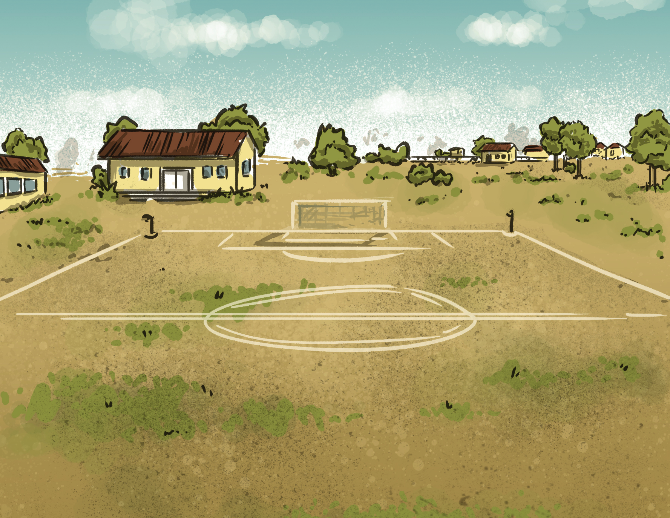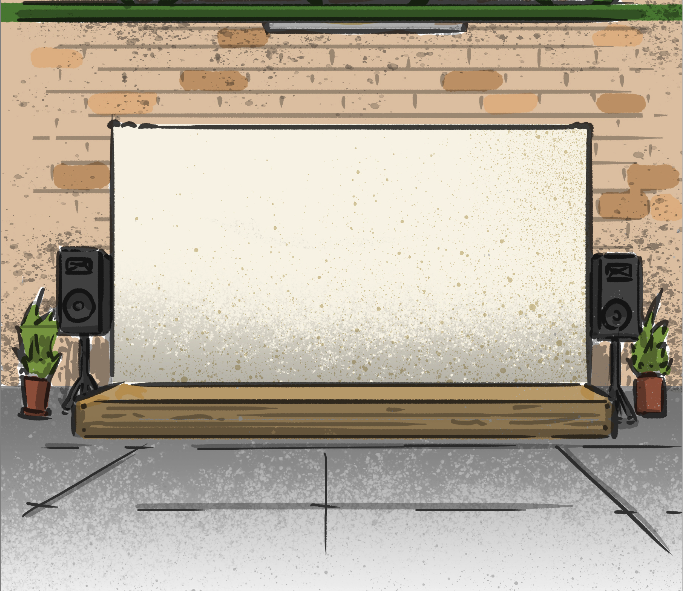  Football pitch Song contest stage  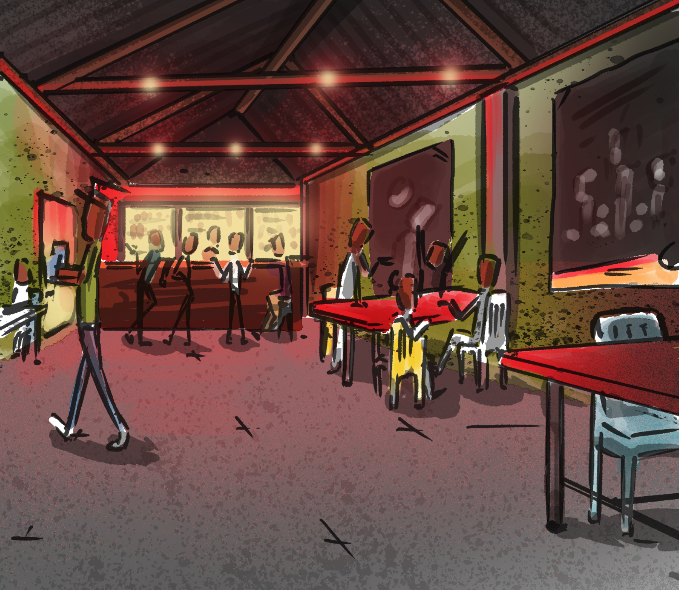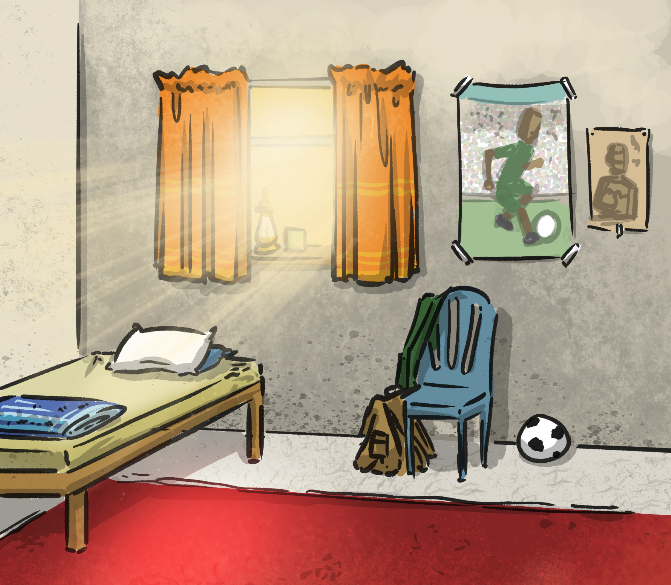  Tavern Main character’s room | |
